# Integrative proteomics reveals MSH6 to modulate PARP inhibitor sensitivity in *BRCA1/2*-proficient ovarian cancer

**DOI:** 10.1371/journal.pone.0357365

**Published:** 2026-09-02

**Authors:** Ou Deng, Thales Da Costa Nepomuceno, Bin Fang, Eric A. Welsh, Victoria Izumi, Rachael H. Martin, Erin M. George, John M. Koomen, Alvaro N. Monteiro, Uwe Rix

**Affiliations:** 1 Department of Drug Discovery, H. Lee Moffitt Cancer Center & Research Institute, Tampa, Florida, United States of America; 2 Department of Cancer Epidemiology, H. Lee Moffitt Cancer Center & Research Institute, Tampa, Florida, United States of America; 3 Proteomics & Metabolomics Core, H. Lee Moffitt Cancer Center & Research Institute, Tampa, Florida, United States of America; 4 Biostatistics and Bioinformatics Shared Resource, H. Lee Moffitt Cancer Center & Research Institute, Tampa, Florida, United States of America; 5 Department of Gynecologic Oncology, H. Lee Moffitt Cancer Center and Research Institute, Tampa, Florida, United States of America; 6 Molecular Oncology, H. Lee Moffitt Cancer Center & Research Institute, Tampa, Florida, United States of America; 7 Department of Molecular Biosciences, University of South Florida, Tampa, Florida, United States of America; University of Salerno, ITALY

## Abstract

Ovarian cancer remains a leading cause of gynecologic cancer–related deaths worldwide. Deficiencies in BRCA1/2 are well-established biomarkers that predict sensitivity to poly(ADP-ribose) polymerase inhibitors (PARPis). However, emerging evidence indicates that a subset of *BRCA*-proficient tumors also responds to PARPi therapy, suggesting the presence of additional molecular mechanisms. We hypothesized that the composition of the PARP1 protein complex and PARylation-mediated signaling contribute to PARPi response in *BRCA*-proficient HGSOC. We assessed PARPi response across a panel of *BRCA*-proficient ovarian cancer cell lines and identified distinct sensitive and resistant groups. Chemical proteomics with rucaparib revealed different PARP1 complexes including higher enrichment of MSH6 in sensitive cells. Co-immunoprecipitation analyses further confirmed differential assembly of PARP1–MSH6–PARP2 complexes between sensitive and resistant models. To explore PARylation signaling, we performed ADP-ribosylation proteomics using clickable NAD⁺ analogs, revealing distinct PARylation profiles between sensitive and resistant cell lines. CHAF1A, a known MSH6 interactor and PARP1 substrate, showed more pronounced reduction in ADP-ribosylation in PARPi-sensitive cells. Targeting *MSH6* using CRISPR or siRNA decreased PARPi sensitivity. In addition, mTOR signaling was reduced in sensitive, but increased in resistant cells, following rucaparib treatment. Notably, *MSH6* knockdown led to increased CHAF1A expression regardless of rucaparib treatment. Importantly, knockdown of *CHAF1A* significantly impaired cell viability, especially in A2780 cells, and suppressed mTOR signaling, suggesting that CHAF1A acts downstream of MSH6 to regulate the mTOR axis. Furthermore, co-treatment with mTORC1 inhibitors enhanced the cellular effects of rucaparib in resistant cells, suggesting a therapeutic potential of targeting downstream mTOR effectors to overcome intrinsic resistance. In conclusion, this study identifies the PARP1–MSH6 interaction to modulate PARPi sensitivity via CHAF1A-mTOR signaling in *BRCA*-proficient ovarian cancer. By integrating chemical proteomics and ADP-ribosylation proteomics, we delineate the interplay between PARP1 complex composition and signaling dynamics, highlighting MSH6 as a critical modulator of PARPi response and potential biomarker to enhance therapeutic efficacy in *BRCA*-proficient HGSOC.

## Introduction

Ovarian cancer (OC) remains one of the most lethal gynecological malignancies [1–3]. Poly(ADP-ribose) polymerase inhibitors (PARPis) have significantly improved outcomes for patients with homologous recombination repair (HR) deficiency (HRD), largely due to loss-of-function mutations in the *BRCA1* and *BRCA2* genes [4–6]. However, emerging evidence indicates that some patients with *BRCA*-proficient ovarian cancers can also benefit from PARPi therapy [7–10]. Although HRD has been implicated in some cases [9,11,12], additional mechanisms have been reported, including replicative gap accumulation [13–15], alterations in DNA damage response signaling [16,17], and *SLFN11* expression [18,19]. Notably, the underlying mechanisms and appropriate biomarkers remain incompletely understood. However, a deeper mechanistic understanding of PARPi sensitivity and inherent resistance in *BRCA*-proficient OC may reveal novel cellular vulnerabilities and drug targets that could be harnessed to develop drug combination therapies that either enhance efficacy in PARPi-sensitive tumors or overcome inherent PARPi-resistance. Furthermore, this may identify additional biomarkers for patients that may be missed by *BRCA*/HRD testing. In our previous work, we have observed that the composition of PARP1-associated protein complexes and patterns of poly-ADP-ribosylation (PARylation) may play critical roles in differentially modulating PARPi response between *BRCA*-deficient and *BRCA*-proficient OC cells [20]. However, despite these insights, comprehensive studies systematically exploring these protein interactions in *BRCA*-proficient OC to differentiate PARPi-sensitive from -resistant tumors are needed.

In this study, we investigated the proteomic landscape associated with PARPi sensitivity in a diverse panel of *BRCA*-proficient OC cell lines, defined here by the absence of *BRCA1/2* mutations or genomic deletions. Most cell lines used in this study have previously been reported to exhibit HR proficiency [21–24]. We applied chemical proteomics and ADP-ribosylation proteomics to identify key PARP1-interacting complexes and downstream PARylation-dependent signaling pathways that correlate with differential drug response. Our findings expand the current understanding of PARPi activity beyond canonical HR deficiency, highlighting novel biomarker candidates and therapeutic vulnerabilities that may inform personalized treatment strategies and broaden the clinical utility of PARPis in ovarian cancer. Preliminary findings from this study were previously presented in abstract form [25].

## Materials and methods

### Cell lines and cell culture

All cell lines were maintained in a humidified incubator at 37 °C with 5% CO_2_. A2780 [26], HEYA8 [27], OVCA433 [28], OVCA432 [28], DOV13, Caov3 [29], OVCAR3, OVCAR8 [30] were cultured in RPMI 1640 with 10% fetal bovine serum (FBS, Gibco). SKOV3 were cultured in ATCC-formulated McCoy's 5a Medium Modified (ATCC 30–2007) with 10% FBS. FU-OV1 [31] and iFTEC282P53R175H were cultured in DMEM/F12 (Wisent) with 10% FBS. UWB+BRCA1 cells were incubated in 1:1 MEBM Bullet Kit and RPMI1640 media (Lonza) supplemented with 3% fetal bovine serum and 200 μg/ml G418 (Life Technologies, Inc). PEO4 were cultured in RPMI 1640 with 10% FBS, 4 mM L-Glutamine and 10 mM HEPES. UWB1.289 + BRCA1 (UWB+BRCA1, ATCC CRL-2946) cell lines and SKOV3 (ATCC HTB-77) were purchased from American Type Culture Collection (ATCC). The OVCAR3 (Cat. No. SCC257) and DOV13 (Cat. No. SCC186) cell lines were obtained from Millipore Sigma. PEO4 was a gift from Dr. Andrew Godwin (University of Kansas Medical Center). A2780, OVCAR8 and HEYA8 were gifts from Dr. Guillermo Armaiz (Ponce Health Sciences University). OVCA433, OVCA432, FU-OV1 and Caov3 were gifts from Dr. Anthony Magliocco (Moffitt Cancer Center). Cell line identity was confirmed by short tandem repeat (STR) profiling prior to experimentation. iFTE282 (Tert-immortalized fallopian tube epithelial cells expressing mutant p53) cells were gift from Simon Gayther (Cedars-Sinai Medical Center), originally generated and described previously [32]. Cells were routinely tested for mycoplasma contamination and confirmed to be negative.

### Compounds

Olaparib (AZD2281; Chemietek), niraparib (MK4827; Chemietek), rucaparib (AG014699; Chemietek), rapamycin (CeMM), Everolimus (CeMM) and LY2584702 (Selleckchem) were dissolved in DMSO at a concentration of 10 mM and stored at −20 °C. 6-alkyne (6Yn)- and 2-alkyne (2Yn)-adenosine probes were synthesized in-house by the chemistry unit of the Chemical Biology Core of the Moffitt Cancer Center according to published procedures [20,33]. Drug dilutions were made in DMSO.

### Immunoblotting

Cells were harvested from culture plates, washed three times with ice-cold PBS, and lysed in buffer containing 0.4% NP40, as previously described [20]. The lysates were precleared twice by centrifugation at 27,000 *x* g at 4 °C for 20 min and protein concentration was determined using standard Bradford assay. Proteins were resolved by SDS-PAGE, transferred to activated polyvinylidene difluoride membranes using the TransBlot Turbo system (Bio-Rad), incubated at 4 °C overnight with primary antibodies, and washed with Tris-buffered saline with Tween-20, followed by 1 h incubation with secondary antibodies at room temperature (RT). Signals were developed with Clarity Western ECL Substrate (Bio-Rad, 1705061) and read on an Odyssey FC Imager using Image studio software (Licor). Antibodies used were against MSH6 (#PIMA532676, Fisher Scientific), PARP1 (#9542S, Cell Signaling), CHAF1A (#5480s, Cell Signaling), mTOR (#2972s, Cell Signaling), phospho-mTOR (pSer2448) (#5536s, Cell Signaling), AKT (#9272S, Cell Signaling), phospho-AKT (pSer473) (#9271S, Cell Signaling), PRAS40 (#2691S, Cell Signaling), phospho-PRAS40 (pThr246) (#13175S, Cell Signaling), S6 (#2217S, Cell Signaling), phospho-S6 (pSer235/236) (#4858S, Cell Signaling), phospho-H2AX (pSer139)(#29380–1-ap, Proteintech) PARP2 (#39743, Active Motif), Cleaved Caspase-3 (Asp175) (#9661, Cell Signaling) and actin (#A5441, Sigma). Secondary antibodies were horseradish peroxidase–conjugated α-rabbit or α-mouse (GE Healthcare).

### Crystal violet cell viability assay

Cells were plated in a 6-well plate at 3,000 cells/well and treated with the appropriate drugs for a total of 10 days, as previously described [20,34]. Cells were fixed with methanol and stained with 0.1% crystal violet solution before imaging using a tabletop scanner. Crystal violet was quantified using methanol extraction and analyzed at 540 nm on a M5 Spectramax plate reader (Molecular Devices). Data were normalized to vehicle-treated wells and fit to a sigmoidal dose-response curve using GraphPad Prism 10 software (GraphPad Software Inc). Drug combination effects were assessed by the Bliss independence model [35].If the combined effect was greater than expected for each drug additively, the response was classified as synergistic (Bliss score >0), while antagonism is concluded when the combination produces less than the expected additive effect (Bliss score <0).

### RNA interference

Small interfering RNAs (siRNAs) targeting MSH6 and CHAF1A, as well as a negative control, were used for gene silencing experiments. The following siRNAs were obtained from Thermo Fisher Scientific: Silencer® Negative Control #1 siRNA (Catalog #AM4611), siMSH6–1 (Silencer™ Pre-Designed siRNA, Assay ID: 144497), siMSH6–2 (Silencer™ Pre-Designed siRNA, Assay ID: 144498), siCHAF1A (Silencer™ Pre-Designed siRNA, Assay ID: 16462). Transfections were performed in 6-well plates according to the manufacturer’s instructions. Knockdown efficiency was assessed by western blotting. Cells were seeded at a density of 3 × 10⁵ cells per well and treated with siRNAs and/or drugs for the indicated time. Following treatment, cells were harvested by trypsinization, washed, and resuspended in 1 mL of RPMI-1640 culture medium. A 20 µL aliquot of each cell suspension was transferred in triplicate into Corning black, flat-bottom 96-well plates. Cell viability was assessed using the CellTiter-Glo® Luminescent Cell Viability Assay (Promega). Remaining cells were collected for immunoblot analysis.

### CRISPR-Cas9-mediated MSH6 knockout

Oligonucleotides targeting the *MSH6* gene were designed using the Benchling CRISPR Genome Engineering tool (https://www.benchling.com) and cloned into the lentiCRISPR v2 vector (Addgene #52961) [36] via the BsmBI restriction site. The resulting plasmids were transformed into **Stbl2** competent cells for amplification. Single bacterial clones were selected and verified by Sanger sequencing using the hU6 forward and EF1α reverse primers. Two guide RNA sequences were used for *MSH6* targeting: **MSH6−1**: Forward: 5′-CACCGGAACATTCATCCGCGAGAA-3′; Reverse: 5′-AAACTTCTCGCGGATGAATGTTCC-3′. **MSH6−2**: Forward: 5′-CACCGCAGGGACGTAACAACCCATC-3′; Reverse: 5′-AAACGATGGGTTGTTACGTCCCTGC-3′. Lentiviral particles were produced in HEK293T cells using the third-generation lentiviral packaging system (Applied Biological Materials, Cat# LV053). Viral supernatants were harvested and filtered to remove cellular debris. Target cells were transduced with 1 mL of viral filtrate supplemented with 4 µg/mL polybrene. After 24 hours, transduced cells were selected using puromycin. Single-cell clones were isolated by limiting dilution in 96-well plates and sequentially expanded into 6-well plates and 10 cm dishes. Successful *MSH6* knockout was confirmed by western blot analysis. A non-targeting control vector (lentiCRISPR v2-sgControl; Addgene #125836) was used as a negative control.

### Chemical proteomics

We have previously described the synthesis and validation of c-rucaparib [37]. Immobilization and drug pull-down experiments were performed as previously described in a step-by-step protocol [20,38] with modifications as follows: c-rucaparib were immobilized on NHS-activated Sepharose beads by overnight RT incubation in the presence of triethylamine. Successful coupling was confirmed using HPLC-MS and beads were blocked overnight with ethanolamine. Lysates (5 mg per sample) were preincubated with competition compound rucaparib (20 μM) or DMSO for 30 min at 4 °C. The drug beads were washed with DMSO followed by lysis buffer and affinity pull-down experiments were performed by incubating drug beads with lysates for 2 h at 4 °C. Beads were further washed on Bio-spin disposable chromatography columns (Bio-Rad) with lysis buffer, bound proteins were eluted by heating to 95 °C in 30 μl of Laemmli buffer for 5 min. A portion of each eluate was set aside for analysis by Western blotting. The eluates were run on SDS-PAGE, followed by in-gel trypsin digestion, and a nanoflow ultra high performance liquid chromatograph and nanoelectrospray orbitrap mass spectrometer (EvoSep and Q-Exactive plus) were used for LC-MS/MS. The sample was loaded onto a EvoTip pure (EV2013). Trapped peptides were eluted onto the analytical column (EV1106, 15 cm length x 150 µm ID, 1.9 µm particle size). A factory default extended gradient (88-minute) was used with solvent A (water + 0.1% formic acid) and solvent B (acetonitrile + 0.1% formic acid). Spray voltage was 1900 V. Capillary temperature was 275 °C. S lens RF level was set at 50. Data-dependent acquisition was performed using Top 16 precursors. The resolution for MS and MS/MS were set at 70,000 and 17,500 respectively. Dynamic exclusion was 15 seconds for previously sampled peaks. MaxQuant [39] (version 1.6.1.14) was used to identify human proteins using the UniProt database (May 2022) and quantify their relative intensities. Up to 2 missed trypsin cleavages were allowed. The mass tolerance was 20 ppm for the first search and 4.5 ppm for the main search. Carbamidomethyl cysteine was set as fixed modification. Methionine oxidation was set as a variable modification. Both peptide spectral match (PSM) and protein false discovery rate (FDR) were set at 0.01. The “match between runs” feature was activated to carry identifications across samples. Similar setting were used for Mascot (Matrix Science) to support data upload to PRIDE/ProteomeXchange [40]. The data were filtered for common contaminants, reverse hits, and intensity value = 0. Missing values were imputed with the lowest value of each column and iBAQ intensities were Log2 transformed. A cutoff of 1 for Log2 ratios of pull downs and competition controls, and p value (<0.05) were applied. The experiment was performed with three biological replicates. Chemical proteomics was performed on six cell lines (three drug-sensitive and three drug-resistant). SKOV3 cells were excluded from further analysis due to inconsistent target enrichment of PARP1.

### ADP-ribosylation proteomics

These experiments were performed as previously described [20,33]. Briefly, the cells were treated with 10 μM rucaparib or DMSO for 1 h, medium was removed, and the cells were treated with 0.5 mM 6YnAd and 0.5 mM 2YnAd for an additional hour. Whole cell lysates were prepared, and total protein was quantified for each sample using the Bradford assay. For each condition, 500 μg of total protein was subjected to click reactions using Azide-PEG3-Biotin or Azide-TAMRA-Biotin capture reagent. Proteins were precipitated using methanol/chloroform/water and air-dried precipitates were resuspended. An aliquot of protein (30 μg) was loaded per gel lane (4%–15% mini-PROTEAN TGX precast gels, Bio-Rad) and resolved by SDS-PAGE. The gels were scanned for fluorescence labeling and read on an Odyssey FC Imager using Image studio software (Licor). 500 μg of protein was subjected to affinity enrichment on NeutrAvidin-agarose beads (Thermo Scientific, Cat# 29200). After extensive washing, the beads were subjected to 3 mM DTT treatment, 10 mM iodoacetamide treatment, and overnight trypsinization. Samples were acidified to pH 3 using formic acid, allowed to stand for 5 min, centrifuged, and the supernatant was collected. Beads were washed with 0.1% formic acid in water, centrifuged, and supernatants were mixed with the previous supernatants. Samples were labeled using TMT reagents as described by the manufacturer (Thermo Fisher TMT 18plex Isobaric Mass Tagging Kit, # A58335). Labeling efficiency was confirmed by LC-MS/MS. Each batch included a pooled DMSO-treated sample from all six cell lines as an internal control for batch normalization. After sample combination and lyophilization, peptides were redissolved in 250 μl of aqueous 20 mM ammonium formate buffer (pH 10.0). Basic pH reversed-phase liquid chromatography separation was performed on an XBridge column (Waters). The concatenated peptide fractions were dried by vacuum centrifugation. A nanoflow ultra high performance liquid chromatograph (RSLC, Thermo) and an electrospray bench top orbitrap mass spectrometer with high field asymmetric waveform ion mobility spectrometry (Orbitrap Exploris 480 with FAIMS, Thermo) were used for tandem mass spectrometry peptide sequencing experiments. The sample was first loaded onto a pre-column (C18 PepMap100, 2 cm x 100 µm ID packed with C18 reversed-phase resin, 5 µm particle size, 100 Å pore size) and washed for 8 minutes with aqueous 2% acetonitrile and 0.04% trifluoroacetic acid. The trapped peptides were eluted onto the analytical column (C18 PepMap100, 75 µm ID x 25 cm, 2 µm particle size, 100 Å pore size, Thermo). The 120-minute gradient was programmed as: 95% solvent A (aqueous 2% acetonitrile + 0.1% formic acid) for 8 minutes, solvent B (aqueous 90% acetonitrile + 0.1% formic acid) from 5% to 38.5% in 90 minutes, then solvent B from 50% to 90% B in 7 minutes and held at 90% for 5 minutes, followed by solvent B from 90% to 5% in 1 minute and re-equilibration for 10 minutes. The flow rate on analytical column was 300 nl/min. Spray voltage was 2100 V, and capillary temperature was 300 °C. Cycle time was set at 1.5 sec for data dependent acquisition for two FAIMS compensation voltage values (−45 and −65). The resolution for MS and MS/MS scans were set at 120,000 and 45,000 respectively. Dynamic exclusion was 15 seconds for previously sampled peptide peaks.

MaxQuant (version 1.6.14.0) [39] was used to identify peptides and quantify the TMT reporter ion intensities. Protein database was downloaded from UniProt in March 2023. Up to 2 missed trypsin cleavages were allowed per peptide. Carbamidomethyl cysteine was set as a fixed modification, and methionine oxidation was set as a variable modification. Both peptide spectral match (PSM) and protein false discovery rate (FDR) were set at 0.01. The “match between runs” feature was activated. The data were then filtered for common contaminants (e.g., non-human proteins, etc.) and reverse sequences. For the analysis and comparison of TMT 18-plex data, the reporter ion intensity was used for the relative quantification of each peptide. IRON (iterative rank-order normalization) of MaxQuant data was performed as described before [41]. The proteins with were filtered by three different criteria: Criterion 1 (C1): Proteins with an average log2 ratio (sensitive vs. resistant cells, DMSO-treated) ≥ 2 standard deviations (SD) and p ≤ 0.05. Criterion 2 (C2): Proteins with a log2 ratio of rucaparib vs. DMSO < 0 and p < 0.05 in individual cell lines. Criterion 3 (C3): Proteins showing a log2 ratio of rucaparib vs. DMSO < 0 in at least two PARPi-sensitive cell lines. The experiment was performed with three biological replicates. Each treated sample was run as two technical replicates.

### Pull-down of substrate proteins of PARP1 using adenosine probes

Cells were treated with or without rucaparib for 1h, followed by treatment with 0.5 mM mixture of the probes. Control experiments were done without probe. Click chemistry was carried out using Azide-TAMRA-Biotin as described before [20,33]. Following protein precipitation, 30 μg of protein was set aside as input control. A total of 500 μg of protein was subjected to affinity enrichment using NeutrAvidin-Agarose beads. The beads were washed once, the beads and input were boiled at 95°C for 5 minutes in 2X loading buffer (Bio-Rad) containing 10% β-mercaptoethanol and proteins were collected by centrifugation. The proteins were resolved by SDS-PAGE on 4%–15% precast gels and scanned for fluorescence labeling using Licor. The input gel was stained with AcquaStain (Bulldog Bio). The gel with enriched proteins was transferred to a nitrocellulose membrane for Western blotting with an anti-CHAF1A antibody.

### Quantification and statistical analysis

Data was obtained from at least three independent experiments and shown as the mean ± SD. Data was analyzed using Microsoft Excel and GraphPad Prism 10.0. Statistical analyses were performed using Student’s t test (paired or unpaired, as appropriate) and synergy was determined by the Bliss independence model [35]. P value of <0.05 was considered statistically significant. All statistical details are included in the figure legends. All MaxQuant data were first filtered for peptides with PEP score < 0.05. Furthermore, reverse and contaminant peptides and peptides with no intensity were excluded. Data were then normalized using IRON [41].

### Gene set enrichment analyses and data visualization

The heatmap of enriched terms across the input of gene lists from chemical proteomics was created by Metascape [42] and colored according to p-values. Protein–protein interaction analysis was carried out with all protein interaction databases using the Metascape [42] and STRING [43].

## Results

### *BRCA*-proficient ovarian cancer cells show a wide range of sensitivity to PARP inhibitors

**Fig 1:**
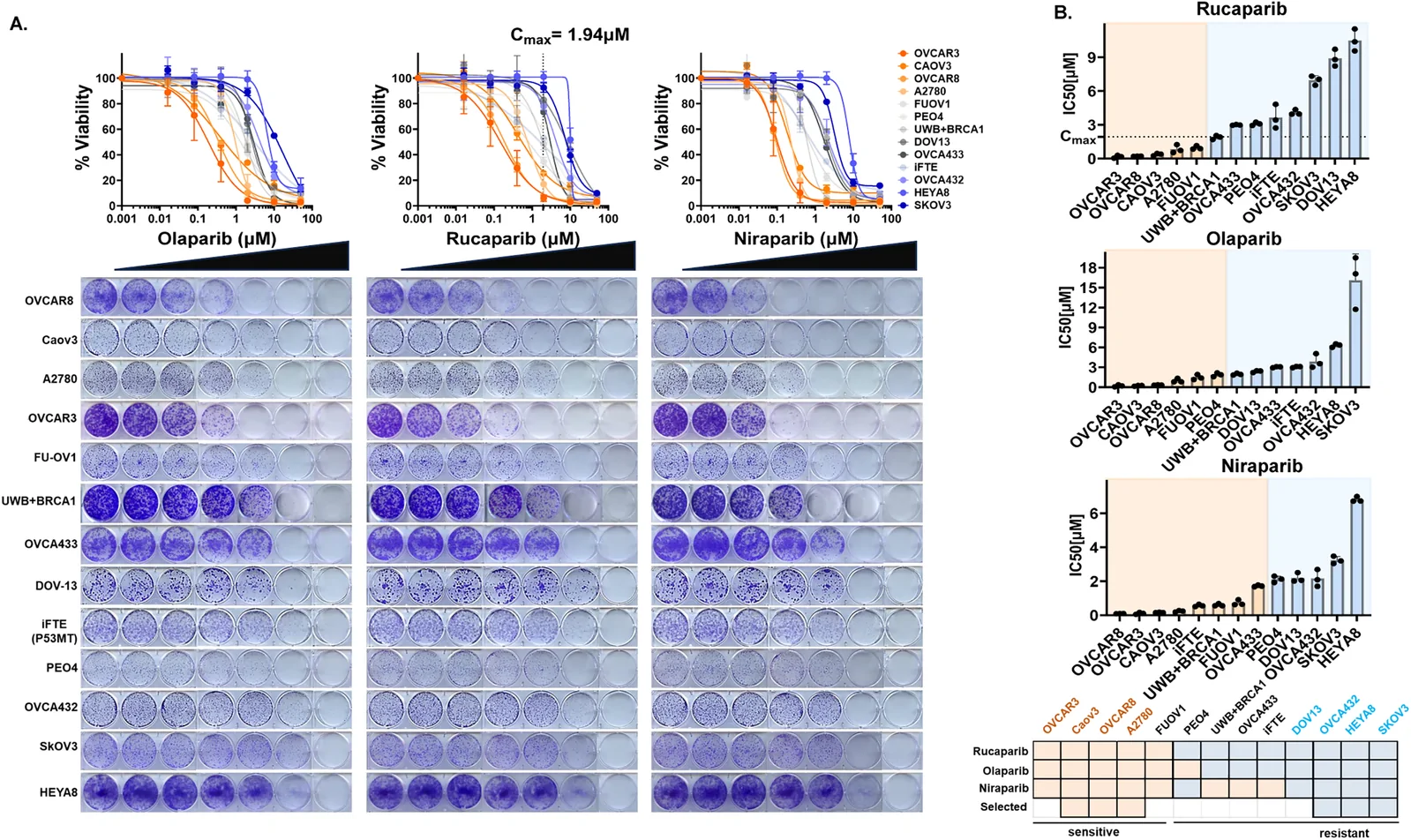
PARPi sensitivity of BRCA-proficient OC cell lines. (A) Crystal violet clonogenic survival assay for a panel of *BRCA*-proficient ovarian cancer cell lines treated with olaparib, rucaparib, or niraparib at concentrations of 0, 0.08, 0.4, 2, 10, 50 μM after treatment for 10 days. Upper panel: quantification of crystal violet intensity; lower panel: representative images of stained wells. Error bars represent the mean ± SD of three biological replicates. (B) Half-maximal inhibitory concentration (IC₅₀) values derived from the dose–response curves shown in panel A, summarizing the relative sensitivity of each cell line to olaparib, rucaparib, and niraparib. The heatmap at the bottom indicates relative PARPi sensitivity based on IC₅₀ values relative to rucaparib Cmax (1.94 μM). Light peach indicates IC₅₀ < Cmax and light blue indicates IC₅₀ > Cmax. Representative PARPi-sensitive and -resistant cell lines were selected based on consistently low or high IC₅₀ rankings across all three PARP inhibitors.

To evaluate the sensitivity of *BRCA*-proficient OC cells to PARPis, we treated 12 *BRCA*-proficient OC cell lines, and one immortalized fallopian tube epithelial (iFTE) cell line expressing mutant p53 [32], with the FDA approved PARPis olaparib, rucaparib and niraparib for 10 days. Clonogenic viability assays revealed that the response to PARPi treatment varied greatly across cell lines, indicating differential sensitivity profiles (Fig 1A). Based on their IC_50_ values, relative to rucaparib’s maximal plasma concentration (1.94 μM), and consistency across the other PARPis olaparib and niraparib, cell lines were classified as sensitive or resistant (Fig 1B). DOV13 cells exhibited resistance to rucaparib and niraparib, but remained relatively sensitive to olaparib. Considering that rucaparib was FDA-approved as maintenance therapy for recurrent OC regardless of *BRCA* mutation status, we focused our analysis primarily on this PARPi. Notably, at its clinically relevant peak plasma concentration (C_max_ = 1.94 μM) [44], only about a third of the tested cell lines exhibited greater than 50% reduction in viability, suggesting that the majority of cell lines were resistant. For subsequent studies, we therefore selected A2780, OVCAR8, and Caov3 as *BRCA*-proficient ovarian cancer cell lines representative for PARPi-sensitivity (Fig 1B). OVCAR3 was excluded despite its sensitivity due to previously reported deep *BRCA2* deletions, although its mRNA expression levels were comparable to those of other OC cell [45]. HEYA8, SKOV3, and OVCA432 were selected as representative *BRCA*-proficient ovarian cancer PARPi-resistant lines.

### PARP1 forms differential protein complexes with PARP2 and MSH6 in PARPi-sensitive and -resistant OC cells

**Fig 2:**
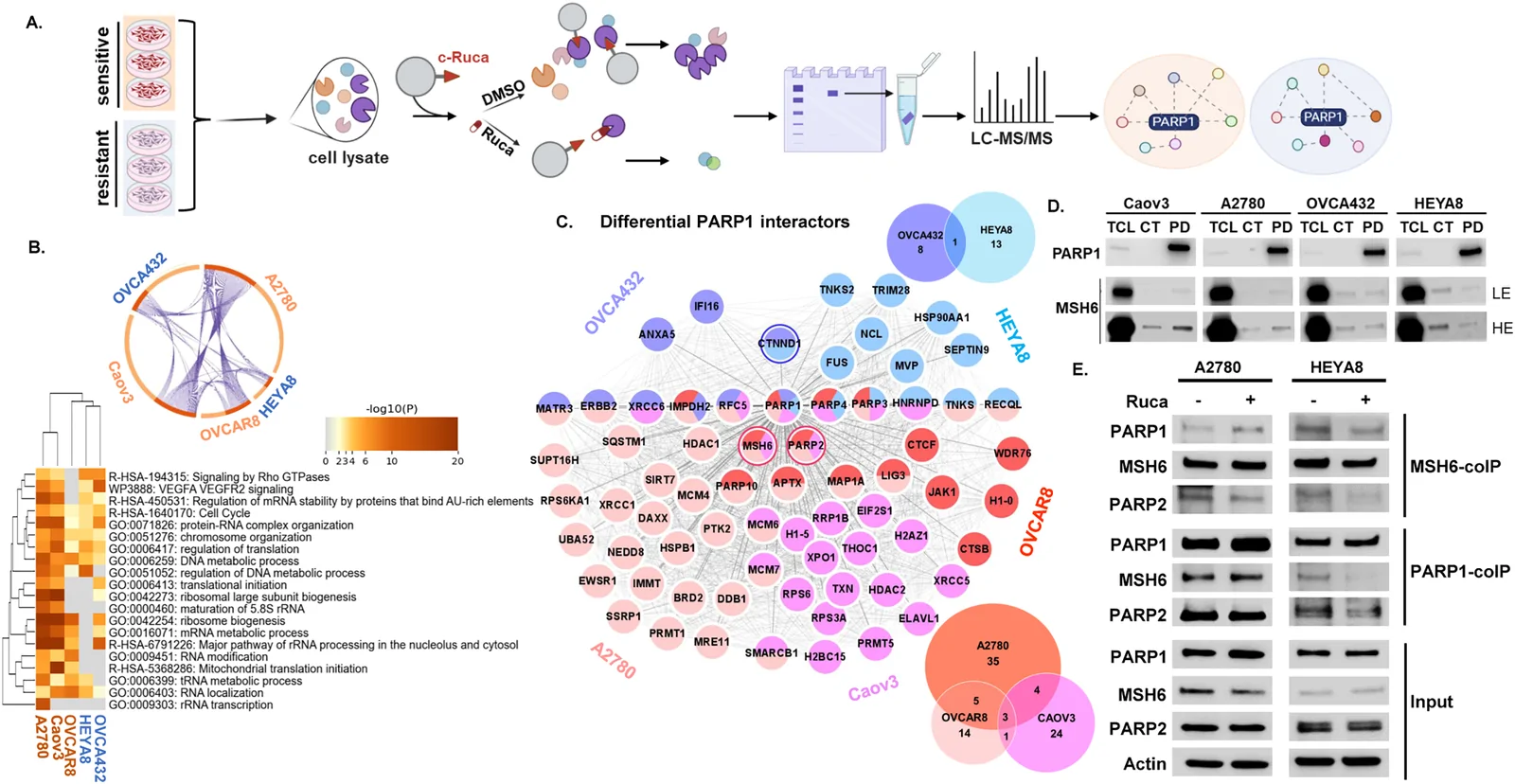
Chemical proteomics with rucaparib in PARPi-sensitive and -resistant *BRCA*-proficient OC cell lines. (A) Workflow of competitive chemical proteomics using rucaparib probe to identify PARP1 and associated interacting proteins in the most PARPi-sensitive and -resistant *BRCA*-proficient OC cell lines. Created in BioRender. Deng, O. (2026) https://BioRender.com/syisc8s. (B) Circos plot (upper panel) of the overlap of enriched interactors identified by rucaparib pulldown compared to competition control across five cell lines. The potential interaction partners were defined by log2 fold change ≥1 and p ≤ 0.05 The heatmap (lower panel) displays differentially enriched ontology clusters by analysis of the potential rucaparib targets across cell lines using Metascape [42] (minimum overlap = 3, p-value ≤ 0.01, minimum enrichment = 1.5). Gray boxes indicate lack of statistical significance. (C) Protein-protein interaction network and differential composition of the PARP1 complex in PARPi-resistant (blue) versus -sensitive (red) cell lines. Protein interaction network was generated using Metascape with input genes selected via STRING [43] (minimum interaction score cutoff = 0.4). Color code for pie sector represents a protein list from different cell lines. Venn diagrams show the number of overlapping potential PARP1 interactors among PARPi-sensitive (lower right) and -resistant (upper right) cell lines. (D) Immunoblot of pulldowns using c-rucaparib affinity beads incubated with lysates from Caov3, A2780, OVCA432, and HEYA8 cells without (PD) or with 20 μM free rucaparib competition (CT). Representative blots from three independent experiments are shown. TCL: total cell lysate. Blots for PARP1 and MSH6 were obtained from the same gel. For MSH6, lower (LE) and higher (HE) exposure images are shown. (E) Western blot analysis of eluates from immunoprecipitation (IP) with MSH6 or PARP1 antibodies. A2780 and HEYA8 cells were treated with 10 μM rucaparib for 1 hour and subjected to co-IP, followed by Western blotting with the indicated antibodies. Input samples were run alongside each co-IP (MSH6 and PARP1) in separate gels.

We previously have reported a chemical proteomics-based affinity purification approach with various clinical PARPis [20,37,46], and furthermore observed differential protein binding partners of PARP1 in *BRCA1*-deficient and *BRCA1-*proficient isogenic OC cell lines [20]. To explore the differential composition of PARP1-based protein complexes between PARPi-sensitive and PARPi-resistant *BRCA*-proficient OC cells, we employed a mass spectrometry (MS)-based chemical proteomics approach using rucaparib as a PARP1 probe (Fig 2A).

We harnessed a previously described rucaparib analogue [37] as affinity bait for chemical proteomics experiments with three different PARPi-sensitive (A2780, Caov3, OVCAR8) and -resistant (SKOV3, OVCA432, HEYA8) OC cell lines. Notably, although this information was not available for OVCA432, cell lines A2780, OVCAR8, Caov3, HEYA8 and SKOV3 have been shown previously to be HR proficient [21–24]. Prior to MS analysis, we confirmed PARP1 enrichment by western blotting (WB) using the rucaparib affinity matrix across three biological replicates. As expected, PARP1 was consistently enriched in all cell lines and was fully competed by free rucaparib, regardless of their PARPi sensitivity (S1A Fig in S1 File). Label-free quantitative LC-MS/MS analysis and subsequent data filtering by comparison with negative control/competition samples derived from rucaparib affinity purifications in the presence of unmodified rucaparib, identified binding of specific drug targets and their interaction partners (Fig 2A and S1 Table). Among the six cell lines analyzed, SKOV3 was excluded due to an anomalously lower PARP1 enrichment in the drug pull-down condition compared to the competition condition.

Importantly, 13 proteins were shared among all three PARPi-sensitive cell lines, whereas 10 proteins were commonly observed across both resistant cell lines (HEYA8 and OVCA432) (S1 Table). Pathway analysis of the potential targets using Metascape [42] revealed enrichment of the cell cycle, protein-RNA complex organization, chromosome organization, regulation of translation and DNA metabolic process across all cell lines (Fig 2B). However, DNA metabolic processes were significantly more enriched in PARPi-sensitive cells compared to resistant ones. In addition, PARP1 protein interaction subnetwork analysis by STRING [43] identified PARP1/CTNND1 complexes to be predominantly enriched in PARPi-resistant cells (Fig 2C). Conversely, the known PARP1 binding partners PARP2 (which is furthermore a direct rucaparib target) and the key mismatch repair protein MSH6 were consistently enriched in PARPi-sensitive cells, but not in resistant cells. Interestingly, PARP3 was enriched in all the PARPi-sensitive cells, but also in the PARPi-resistant HEYA8 cells.

Notably, immunoblotting of PARP1-associated interactors further confirmed a co-enrichment of MSH6 in rucaparib pulldowns. Comparison with each own rucaparib competition control, which is critical for distinguishing specific interactions from background, moreover indicated that this interaction was specific in the PARPi-sensitive Caov3 and A2780 cells, but not in the PARPi-resistant OVCAR432 and HEYA8 cells where MSH6 was not competed (Fig 2D). Furthermore, co-immunoprecipitation (co-IP) experiments revealed that the PARP1/MSH6 and PARP1/PARP2 complexes were more abundant in A2780 cells compared to HEYA8 cells (Fig 2E, S1B Fig in S1 File). Rucaparib treatment slightly enhanced PARP1/MSH6 complex formation in A2780 cells but reduced it in HEYA8 cells, suggesting that PARP1/MSH6 interaction is more pronounced in PARPi-sensitive OC cells. Whereas PARP2 was co-immunoprecipitated by PARP1 in both cell lines, this was also observed upon MSH6 co-IP suggesting a shared protein complex of these three proteins. In contrast to MSH6, however, rucaparib treatment reduced the amount of co-immunoprecipitated PARP2 similarly in both cell lines.

Taken together, chemical proteomics with rucaparib revealed that PARP1/PARP2/MSH6 complexes are preferentially enriched in PARPi-sensitive ovarian cancer cells. These findings highlight distinct PARP1-associated protein networks that may contribute to differential PARPi sensitivity in ovarian cancer.

### CHAF1A is ADP-ribosylated and preferentially interacts with PARP1 and MSH6 in PARPi-sensitive OC cells

**Fig 3:**
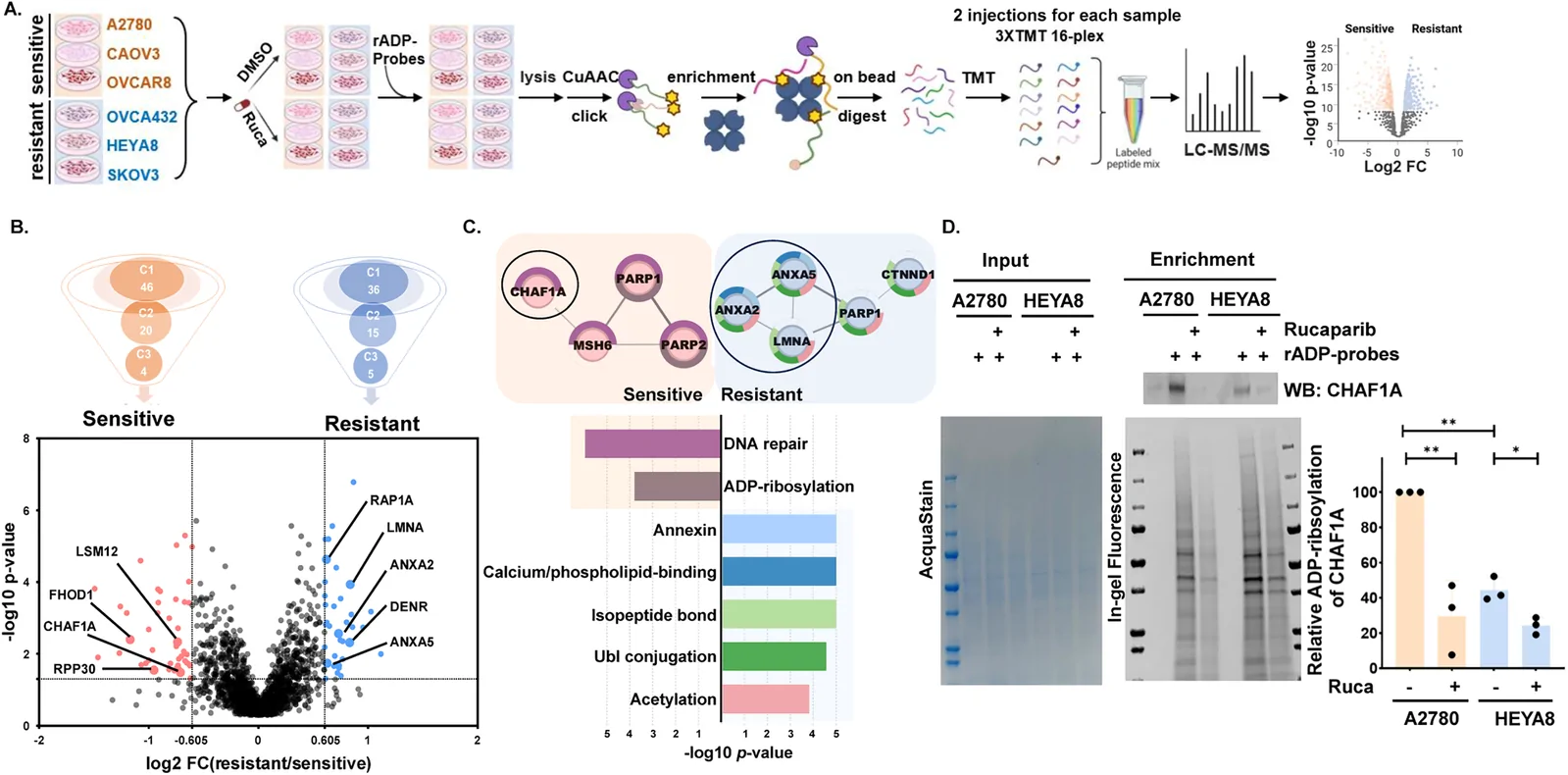
Proteome-wide identification of ADP-ribosylation profile and signaling in response to rucaparib in *BRCA*-proficient OC cells. (A) Schematic of the experimental workflow for identification of ADP-ribosylated proteins. Created in BioRender. Deng, O. (2026) https://BioRender.com/syisc8s. Indicated PARPi-sensitive and -resistant cell lines were treated with or without rucaparib (10 μM) for 1 hour followed by labeling with a mixture of 6-alkyne adenosine and 2-alkyne adenosine probes (0.5 mM each) for 1 hour. CuAAC: copper(I)-catalyzed azide-alkyne cycloaddition. (B) Upper panel: funnel diagrams of the prioritization workflow based on the three selection criteria. Lower panel: volcano plots of the differential enrichment of ADP-ribosylated endogenous proteins between PARPi-sensitive and -resistant cell lines in DMSO-treated samples. Proteins meeting selection threshold C1 are highlighted in pink (enriched in sensitive cells) or blue (enriched in resistant cells). Proteins that also meet criteria C2 and C3, representing consistent inhibition by rucaparib in at least two sensitive or resistant cell lines, are labeled and indicated by larger dot sizes. Detailed criteria are described in the Methods. (C) Integrated analysis of selected significantly modulated proteins identified by chemical proteomics and ADP-ribosylation proteomics in PARPi-sensitive (left panel, red) and -resistant cells (right panel, blue). Proteins identified through ADP-ribosylation proteomics are marked with black circles. Proteins were mapped to STRING enrichment with uniport keyword categorization [43], a false discovery rate (FDR) ≤ 0.05 and minimum interaction confidence score of 0.4. (D) Upper panel: Western blot analysis for CHAF1A of enriched ADP-ribosylation eluates from PARPi-sensitive A2780 and PARPi-resistant HEYA8 cells, with quantification of CHAF1A shown in the lower right, error bars represent mean ± SD of three biological replicates. Cells were treated with or without rucaparib, followed by probe labeling. Lower panel: qualitative assessment of probe labeling in both cell lines after enrichment by total input protein profile stained with Coomassie Blue prior to enrichment (left) and in-gel fluorescence scanning after the enrichment (middle).

Considering that some of the observed PARP1 interactions were modulated by treatment with the PARPi, rucaparib, it was possible that these interactions were governed by PARylation. To identify differential ADP-ribosylation patterns and associated signaling events in response to rucaparib treatment between PARPi-sensitive and -insensitive cells, we performed ADP-ribosylation proteomics using a previously described metabolic labeling strategy with 6-alkyne (6Yn)- and 2-alkyne (2Yn)-adenosines probes, which i*n situ* are incorporated in ADP ribose [20,33]. Cells were treated with rucaparib or DMSO followed by 6/2Yn-adenosine probes. Copper-catalyzed click chemistry labeled proteins with biotin which enabled enrichment using NeutrAvidin-agarose beads (Fig 3A). In-gel fluorescence imaging confirmed significant enrichment of ADP-ribosylated proteins in the probe-labeled samples, which was markedly reduced by rucaparib treatment (S2A Fig). Quantitative proteomics was performed using tandem mass tags (TMT) and quantified by LC-MS/MS. Following data filtering and normalization, 1856 proteins were quantified in this TMT experiment (S2 Table). Comparative statistical analysis between PARPi-sensitive and -resistant cells (under DMSO conditions) revealed 46 and 36 proteins preferentially ADP-ribosylated in sensitive and resistant cells, respectively, which had an average log_2_ ratio (sensitive vs. resistant) ≥ 2 standard deviations and p ≤ 0.05 (criterion C1) (Fig 3B). Further data filtering by criteria C2 (log2 ratio of rucaparib vs. DMSO < 0 and p < 0.05) and C3 (log2 ratio of rucaparib vs. DMSO < 0 in at least two PARPi-sensitive cell lines) highlighted four proteins in sensitive cells and five in resistant cells that exhibited reduced signals in ADP-ribosylation upon rucaparib treatment. Specifically, these targets were CHAF1A, LSM12, RPP30 and FHOD1 in PARPi-sensitive cells, and LMNA, DENR, ANXA2, ANXA5 and RAP1A in -resistant cells (Fig 3B and Fig S2B in S1 File).

To explore functional relevance, we performed protein-protein interaction enrichment analysis using STRING [43] by integrating ADP-ribosylation proteomics and PARP1 interactors from chemical proteomics. This analysis revealed CHAF1A, MSH6, PARP1 and PARP2 complexes and a preferential enrichment for DNA repair in PARPi-sensitive cells. In contrast, enrichment in resistant cells indicated CTNND1, RAP1A, ANXA5, ANXA2 and PARP1 complexes, which involved annexin, calcium/phospholipid-binding, isopeptide bond, Ubl conjugation and acetylation pathways (Fig 3C). Although MSH6 was not one of the proteins detected by PARylation proteomics, MSH6 has been reported to interact with CHAF1A (also known as CAF-1 p150) [47], a subunit of the chromatin assembly factor 1 complex, which was found to be ADP-ribosylated in sensitive cells (Fig 3B). Subsequent immunoblot analysis of ADP-ribosylation pulldowns confirmed ADP-ribosylation of CHAF1A in both A2780 and HEYA8 cells as it was enriched in the probe-labeled samples compared to unlabeled controls and reduced upon treatment with the PARPi rucaparib (Fig 3D). Consistent with the MS-based analysis, CHAF1A was furthermore preferentially ADP-ribosylated in A2780 cells compared to HEYA8 cells.

PARP1 facilitates repair by PARylating target proteins, thereby leading to chromatin relaxation and recruitment of DNA repair factors [48–50]. This premise led us to test if CHAF1A and PARP1/MSH6 form complexes. Co-IP of MSH6 revealed that MSH6 interacted with both PARP1 and CHAF1A and that rucaparib treatment further enhanced the PARP1/MSH6/CHAF1A complexes in A2780 cells, but reduced their enrichment in HEYA8 cells (**Fig S2C** in S1 File), suggesting that PARP1/MSH6/CHAF1A interactions are more pronounced in PARPi-sensitive OC cells, particularly in response to rucaparib treatment. This result furthermore indicated that, whereas CHAF1A was indeed PARylated, the interaction of PARP1, MSH6 and CHAF1A in A2780 cells itself was not dependent of PARylation. In summary, these data show different global ADP-ribosylation landscapes between PARPi-sensitive and -insensitive OC cells and suggest that PARylation of CHAF1A, which participates in the PARP1/MSH6 complex, is downregulated by rucaparib specifically in PARPi-sensitive OC cells.

### MSH6 expression correlates with drug sensitivity

**Fig 4:**
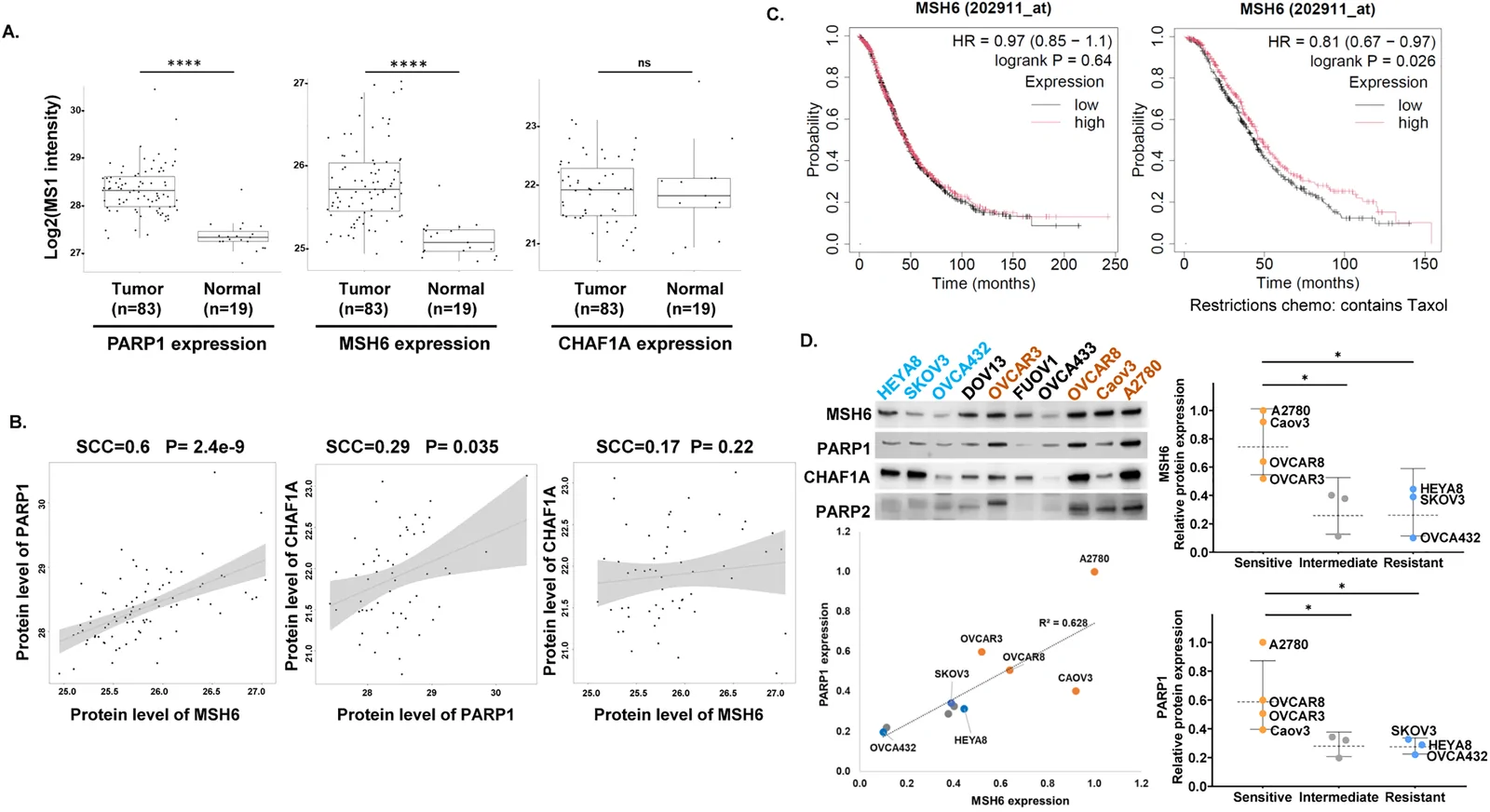
Relationship between MSH6/PARP1/CHAF1A expression and overall survival. (A) Protein expression analysis of PARP1, MSH6, and CHAF1A using CPTAC data accessed through the LinkedOmicsKB web portal [44], comparing primary ovarian tumors and normal ovarian tissues. (B) Protein expression correlation analysis among MSH6, PARP1, and CHAF1A in OC. Scatter plots and correlation between PARP1 and MSH6 (left panel), PARP1 and CHAF1A (middle panel), and MSH6 and CHAF1A (right panel). SCC: Spearman correlation coefficient. (C) Prognostic role of gene expression of *MSH6* in OC patients. Kaplan–Meier plot [51] showing *MSH6* (left panel) is not associated with OS in OC; however *MSH6* expression is associated with OS in the chemotherapy with taxol treatment groups (right panel). (D) Western blot analysis of protein expression levels in *BRCA*-proficient OC cell lines. Quantified protein levels for MSH6 and PARP1 are presented on the right, with each dot representing the mean of three biological replicates per cell line. Erro bars indicate the mean ± SD for each group (sensitive, intermediate and resistant). Protein expression values were normalized to those of the A2780. The lower left panel displays the correlation analysis between MSH6 and PARP1 expression across OC cell lines.

We observed that the PARP1/MSH6/CHAF1A complex is preferentially formed in PARPi-sensitive *BRCA*-proficient OC cells. To investigate the functional role of PARP1, MSH6 and CHAF1A in modulating the PARPi sensitivity of OC cells, we next assessed their protein expression levels using the LinkedOmicsKB web portal [44]. Analysis of Clinical Proteomic Tumor Analysis Consortium (CPTAC) proteomic data revealed that both MSH6 and PARP1 protein levels were significantly upregulated in ovarian tumor tissues compared to normal ovarian tissues (Fig 4A). In contrast, CHAF1A did not show significant differential expression. Interestingly, PARP2 expression was relatively low compared to other cancer types and showed no significant difference between normal tissues and ovarian cancer (Fig S3A in S1 File). We next examined whether expression of these proteins was correlated in ovarian tumors. This analysis indicated that PARP1 protein abundance was significantly positively correlated with MSH6 expression (Fig 4B). However, with CHAF1A, PARP1 only showed a weak correlation, and no correlation was observed between MSH6 and CHAF1A protein expression.

To evaluate the prognostic significance of MSH6 and PARP1 in OC, we next performed a survival analysis using online Kaplan-Meier plotter [51], which indicated that *MSH6*, *PARP1* and *CHAF1A* gene expression levels were not significantly associated with overall survival (OS) in ovarian cancer patients (Fig 4C left panel, Fig S3B in S1 File). However, stratified analysis revealed that in patients receiving chemotherapy regimens, including taxol (Fig 4C right panel), platinum (Fig S3C left panel in S1 File), taxol + platinum (Fig S3C right panel in S1 File), elevated *MSH6* expression was significantly associated with better OS. These results suggest that MSH6 expression may be predictive of chemotherapy response. Additionally, Western blot analysis of a panel of *BRCA*-proficient OC cells demonstrated that the protein expression levels of PARP1, MSH6, and PARP2 were markedly higher in PARPi-sensitive ovarian cancer cell lines, particularly OVCAR3, A2780, Caov3, and OVCAR8, compared to less sensitive lines (Fig 4D). In contrast, CHAF1A expression did not correlate with the expression of MSH6 protein or PARPi sensitivity (Fig S3D in S1 File).

These results highlight both, PARP1 and MSH6, as potential biomarker candidates for drug response in ovarian cancer, particularly in the context of PARP inhibitor sensitivity and chemotherapy-based treatment regimens.

### *MSH6* silencing leads to PARP inhibitor resistance

**Fig 5:**
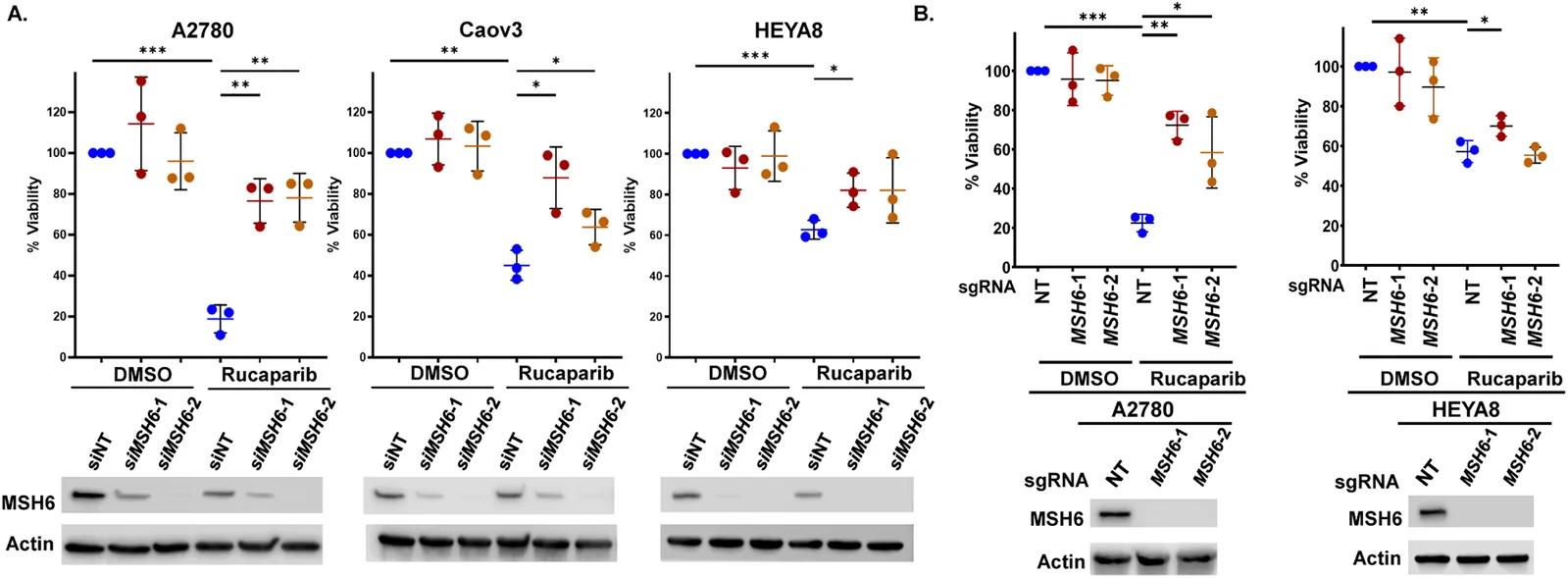
Effect of MSH6 on cell viability. (A, B) Effects of *MSH6* targeting by siRNA-based gene silencing (A) in PARPi-sensitive A2780 cells, Caov3 cells and PARPi-resistant HEYA8 cells, or by CRISPR-based gene knockout (B) in A2780 cells and HEYA8 cells, with or without 10 μM rucaparib treatment for 5 days. Cell viability was measured by CellTiter-Glo (CTG viability) assays. NT: non-targeting control.

To investigate the functional role of MSH6 in OC cells, we next assessed its impact on PARPi response in PARPi-sensitive and -resistant *BRCA*-proficient OC cells. Genetic silencing of *MSH6* using two independent siRNAs had no significant effect on cell survival alone, but significantly rescued cell viability in response to rucaparib treatment (Fig 5A). Although this effect was to some extent observed also in PARPi-resistant HEYA8 cells, it was markedly more pronounced in A2780 and Caov3 cells as these cell lines were much more sensitive to PARPi treatment to begin with. Consistently, CRISPR-Cas9-based gene knockout of *MSH6* strongly rescued A2780 cells from rucaparib treatment and only marginally affected PARPi response of HEYA8 cells (Fig 5B). These results suggested that MSH6 promotes PARPi sensitivity in *BRCA*-proficient OC cells and that low protein levels or loss of MSH6 causes PARPi resistance.

### Loss of MSH6 induces mTOR survival signaling

**Fig 6:**
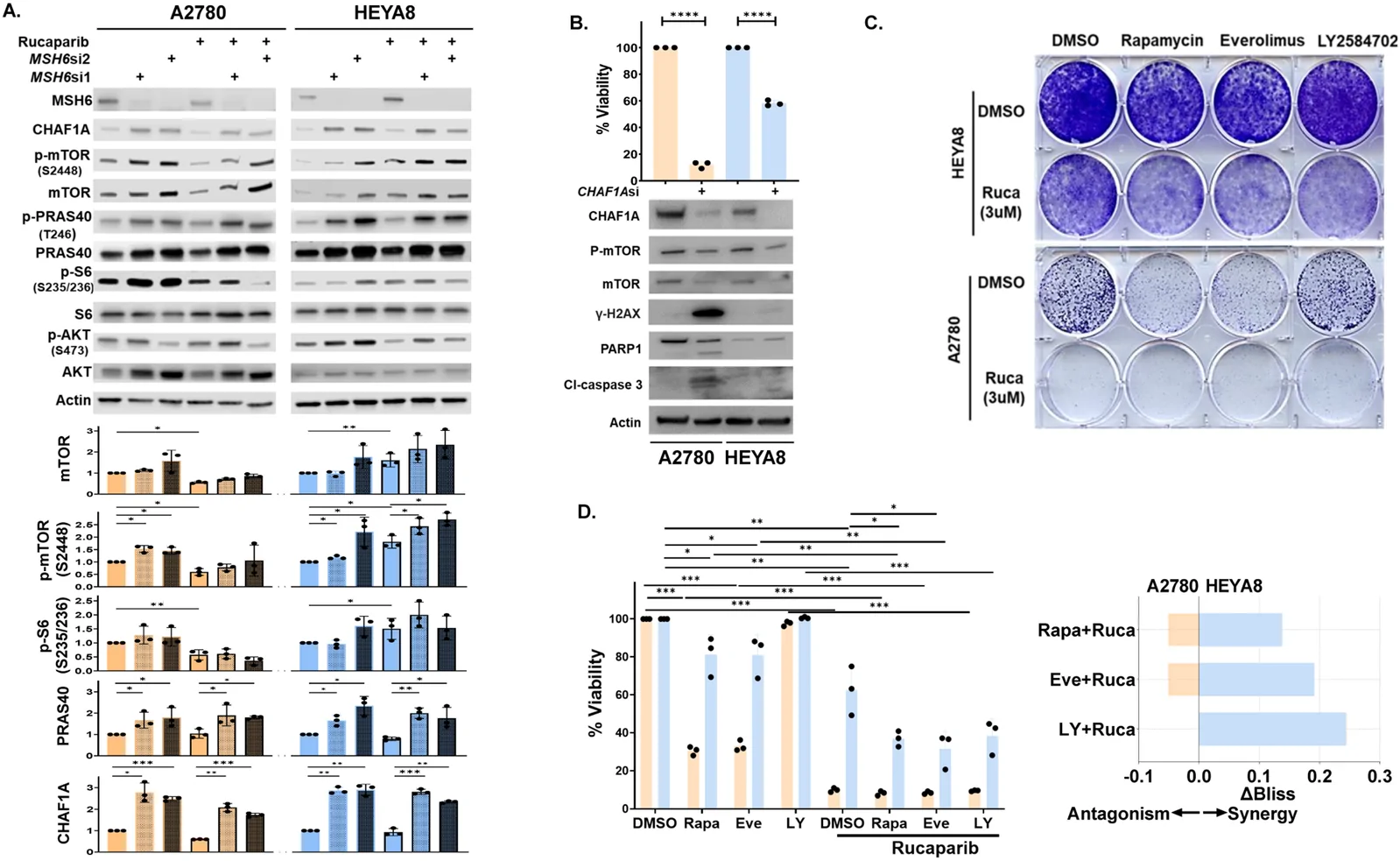
Combination effects of co-targeting PARP1 and mTOR in OC cells. (A) Western blot analysis of A2780 (left) and HEYA8 (right) cells following *MSH6* knockdown via siRNA and treatment with either DMSO or 10 μM rucaparib for 36 hours, with quantification shown on the bottom. (B) Upper panel: Effects of *CHAF1A* targeting by siRNA silencing for 3 days on cell viability of A2780 and HEYA8 cells as assessed by CTG assay. Lower panel: Western blot analysis of the effects of *CHAF1A* knockdown by siRNA on cleavage of PARP1 and caspase 3, on γH2AX, and on mTOR signaling. (C) Clonogenic survival assays of A2780 and HEYA8 cells treated with rucaparib (3 μM) alone or in combination with 0.5 μM mTOR inhibitors (rapamycin, everolimus) or the S6 kinase inhibitor LY2584702 for 7 days as determined by crystal violet staining. (D) Quantification of crystal violet staining intensity from C (left panel), and synergy as determined using ΔBliss (right panel). Positive ΔBliss values indicate synergy, negative ΔBliss values antagonism. Error bars represent mean ± SD of three biological replicates. Ruca: Rucaparib; Rapa: rapamycin; Eve: everolimus; LY: LY2584702.

To explore the underlying mechanism of how MSH6 is promoting PARPi sensitivity, we next queried PARP1 and MSH6 protein expression and determined their associations with pathways using LinkedOmicsKB [44]. This analysis revealed that the protein abundance of both PARP1 and MSH6 were strongly anti-correlated with the KINASE-PSP_Akt1/AKT1 pathway in ovarian tumor samples (Fig S4A and B in S1 File). In addition, this identified AKT1S1 (aka PRAS40) T246, a canonical substrate of AKT1 critical for downstream regulation of mTOR when part of the mTORC1 complex [52,53], as the top associated phosphosite suggesting a link between MSH6 and mTOR signaling (Fig S4C in S1 File). Therefore, we next examined the effects of *MSH6* targeting and rucaparib treatment on the mTORC1 pathway. We knocked down the *MSH6* gene by two different siRNA in PARPi-sensitive A2780 and PARPi-resistant HEYA8 cells and then treated the cells with vehicle or rucaparib (10 μM for 36 hrs). In A2780 cells, rucaparib treatment alone led to reduced levels of mTOR, p-mTOR (Ser2448), and downstream phosphorylation of p-S6 (Ser235/236) (Fig 6A, S4D and S4E in S1 File). In contrast, HEYA8 cells displayed increased levels of these signaling components following rucaparib exposure, suggesting that mTORC1 pathway activation may contribute to intrinsic resistance to PARPi. Notably, *MSH6* knockdown enhanced phosphorylation of p-mTOR (Ser2448), as well as the expression and phosphorylation of PRAS40 upstream of mTORC1 in both cell lines. An increase in p-AKT (Ser473) levels was also observed in rucaparib-treated HEYA8 cells upon *MSH6* depletion, whereas this effect was not observed in A2780 cells. These findings suggest that loss of MSH6 promotes compensatory activation of mTOR-dependent survival signaling, providing a potential mechanism through which MSH6 depletion contributes to reduced PARPi sensitivity.

Interestingly, *MSH6* knockdown also increased total CHAF1A expression in both cell lines, independent of rucaparib treatment. Consistently, silencing of *CHAF1A* significantly inhibited cell survival, which was especially pronounced in the PARPi-sensitive A2780 cells (Fig 6B). *CHAF1A* knockdown also strongly induced apoptosis and DNA damage as evidenced by cleavage of caspase 3 and PARP1, and phosphorylation of H2AX, respectively, in the PARPi-sensitive A2780 cells, whereas this effect was barely discernible in the PARPi-resistant HEYA8 cells (Fig 6B). *CHAF1A* knockdown furthermore attenuated mTOR signaling, primarily by reducing mTOR protein levels, suggesting that CHAF1A acts downstream of MSH6 to regulate the mTOR signaling axis.

Finally, we tested whether mTOR pathway inhibition enhances PARPi efficacy. Combination of rucaparib with either of the two mTORC1 inhibitors rapamycin or everolimus showed greater synergy in HEYA8 compared to A2780 compared to single agent rucaparib, as A2780 cells were much more sensitive to rucaparib to begin with (Fig 6C and D). Consistently, whereas the S6 kinase inhibitor LY2584702 alone had minimal effects, when combined with rucaparib it led to enhanced efficacy in HEYA8 cells.

Together, these findings identify a role for MSH6 in promoting PARPi sensitivity through CHAF1A-mediated regulation of mTORC1 signaling and highlight mTOR activation as a key mechanism of intrinsic PARPi resistance in *BRCA*-proficient ovarian cancer.

## Discussion

Overall, our findings support a model in which PARPi-sensitive and PARPi-resistant BRCA-proficient OC cells exhibit distinct PARP1-associated protein complexes and downstream signaling. PARPi-sensitive compared to PARPi-resistant cells preferentially featured a PARP1-MSH6-CHAF1A complex and higher CHAF1A ADP-ribosylation, reduced by PARPi treatment, which prevented cells to launch PARPi-adaptive mTOR survival signaling. In contrast, PARPi-resistant cells showed compensatory activation of mTOR signaling following PARPi treatment and enhancement of rucaparib effects by pharmacological inhibition of the mTOR pathway. These findings suggest that compensatory mTOR signaling contributes to PARPi resistance and that mTOR pathway inhibition may be a potential combination strategy for PARPi-resistant cells.

PARPis have shown significant clinical benefit in *BRCA*-mutant OC. However, their efficacy in *BRCA*-proficient ovarian tumors remains variable and underexplored. Our study demonstrates that among *BRCA*-proficient OC cell lines, PARPi sensitivity is heterogeneous and not strictly correlated with *BRCA1/2* and *TP53* mutation status, suggesting the involvement of alternative molecular mechanisms [54]. To further investigate the mechanisms underlying PARPi sensitivity and resistance, we employed chemical proteomics and ADP-ribosylation proteomics to examine PARP1 protein complexes and PARylation profiles in PARPi-sensitive and -resistant *BRCA*-proficient OC cells, including the endometrioid OC cell line A2780.

Our proteomic analysis revealed a previously unrecognized PARP1/MSH6/CHAF1A complex enriched in PARPi-sensitive *BRCA*-proficient cells. Although PARP1 and MSH6 have been previously reported to interact during mismatch repair (MMR) [55,56], particularly during the recognition and signaling of DNA mismatches, our chemical proteomics data demonstrated selective enrichment of PARP1/MSH6 complexes in PARPi-sensitive cells with no apparent enrichment of MSH2. This finding suggests an unconventional, potentially MMR-independent role for MSH6 in modulating PARPi sensitivity. MSH6 has been extensively studied in the context of MMR deficiency and microsatellite instability (MSI) [57–60]. However, MSI-high tumors are relatively uncommon in ovarian cancer, occurring in approximately 1–3% of serous cases [61–65]. Although MSI status was not directly assessed in the present study, previous reports have classified SKOV3 as MSI-H, whereas the remaining cell lines used in this study are generally considered microsatellite stable (MSS) [66,67]. Therefore, the differential MSH6 expression and PARP1/MSH6 complex formation observed in our models are unlikely to be explained solely by classical MMR deficiency. Instead, these findings support the possibility that MSH6 may have additional functions beyond canonical mismatch repair, potentially through PARP1-associated signaling pathways that influence PARPi response. The relationship between MSH6-associated signaling, MSI phenotypes, and PARPi sensitivity warrants further investigation.

Notably, rucaparib treatment enhanced the enrichment of the PARP1/MSH6/CHAF1A complex in sensitive cells, whereas this complex was dissociated in resistant cells. The interaction between CHAF1A and MSH6 has previously been shown to be phosphorylation-dependent in that only the dephosphorylated form binds MSH6 [47]. In our study, we determined ADP-ribosylation of CHAF1A, which was significantly enriched in PARPi-sensitive cells and reduced upon rucaparib treatment. These findings support a model in which PARP1 directly modifies CHAF1A via ADP-ribosylation, which may also affect phosphorylation, thereby regulating its interaction preferences and functional activity. These findings indicate that CHAF1A ADP-ribosylation is associated with PARPi response and may contribute to the differential assembly of the PARP1/MSH6/CHAF1A complex.

However, whether CHAF1A is directly ADP-ribosylated by PARP1 or other known PARP family targets, such as PARP2 or tankyrase [37,68], ADP-ribosylation occurs at specific functional sites or contributes directly to CHAF1A’s regulatory activity requires further studies using *PARP1* knockdown, site-directed mutagenesis and mapping of CHAF1A PARylation sites.

CHAF1A is a chromatin assembly factor implicated in replication stress resolution and nucleosome assembly at sites of transcription-replication conflicts (TRCs), which are exacerbated by PARPi treatment [69–71]. In drug-sensitive cells, PARP1-mediated PARylation of CHAF1A was inhibited and may prevent its phosphorylation-dependent recruitment to PCNA [72], instead favoring association with MSH6 after rucaparib treatment. This sequestration may reduce CHAF1A’s role in chromatin reassembly and promote replication stress, contributing to PARPi sensitivity. However, the functional consequences of CHAF1A ADP-ribosylation, its impact on protein interactions and PARPi response were not examined in this study and will require further investigation.

Importantly, we discovered a link between MSH6 and mTOR signaling. Integrative pathway analysis from CPTAC revealed a strong correlation between MSH6 expression and the AKT1/mTOR axis in ovarian tumors. Functionally, rucaparib treatment reduced mTOR signaling in MSH6-high sensitive cells, while *MSH6* knockdown increased phosphorylation of mTORC1 signaling pathway components in both sensitive and resistant cells. *MSH6* silencing also rescued PARPi-induced cell death, suggesting that loss of MSH6 promotes PARPi resistance via upregulation of mTOR signaling.

Interestingly, CHAF1A levels were upregulated following *MSH6* silencing and appeared to act downstream of MSH6 to sustain mTOR signaling. *CHAF1A* knockdown reduced mTOR signaling, induced apoptosis and increased DNA damage particularly in sensitive A2780 cells, indicating its critical role in mediating mTOR-driven resistance.

These findings link MSH6, probably through modulation of CHAF1A–mTOR signaling, to PARPi resistance in *BRCA*-proficient OC. However, whether and how CHAF1A is directly regulated by MSH6 or serves as a critical mediator connecting MSH6 loss to mTOR activation remains to be determined. Consistently, we observed that combination with mTORC1 inhibitors (rapamycin or everolimus) or the S6 inhibitor LY2584702 synergized with rucaparib in HEYA8 cells, where mTOR signaling was induced by rucaparib. This highlights the therapeutic value of targeting downstream mTOR effectors to overcome intrinsic resistance.

In conclusion, using an integrative proteomics approach, we identify a novel PARP1–MSH6 interaction that regulates PARPi sensitivity through modulation of CHAF1A-mTOR signaling in *BRCA*-proficient ovarian cancer cells. Our findings suggest that MSH6 impairs PARPi resistance through suppressing CHAF1A-mediated induction of mTOR signaling, and that MSH6 expression or the interaction of PARP1 with MSH6 may serve as potential predictive biomarker candidates for PARPi therapy and mTORC1 inhibitors. These insights support combination PARPi with mTOR inhibition as a promising strategy to overcome intrinsic resistance in *BRCA*-proficient OC. Furthermore, CHAF1A's dual roles in chromatin regulation and mTOR signaling position it as a potential therapeutic target to overcome PARPi resistance. Because this study is based on integrative proteomics analyses, our conclusions are derived from protein abundance, interactions and signaling changes. Protein and mRNA expression do not always correlate well [73,74], but we do not rule out transcriptional mechanisms.

Although our data identify the interactions among PARP1, MSH6 and CHAF1A and illustrate the functional effects of MSH6 on CHAF1A protein levels and PARPi sensitivity, the precise mechanistic relationship among these proteins remains to be defined. It is currently unclear whether MSH6 directly regulates CHAF1A activity or whether CHAF1A also contributes to modulating PARPi response. Further functional studies, including rescue experiments and targeted perturbation approaches, will be required to clarify these signaling mechanisms in detail. A limitation of the present study is that it is primarily based on established ovarian cancer cell line models. Therefore, further validation experiments will also have to be performed in patient-derived,xenografts (PDX) and organoids (PDO), as well as clinical tumor specimens to establish its translational relevance and potential clinical applicability. In particular, evaluation of MSH6/PARP1 co-expression in larger clinical cohorts will be required to determine its potential utility as a predictive biomarker for PARPi response.

Future studies would be required to validate these findings in vivo and explore the role of post-translational modifications in regulating CHAF1A function. Clinical trials combining PARPis with mTOR or S6K inhibitors in *BRCA*-proficient ovarian cancer may offer a promising strategy to expand the benefit of PARP inhibitors to a broader patient population.

## Supporting information

S1 File Figures S1–S4. (PDF)

S1 Fig Original uncropped and unadjusted blot/gel images underlying the results reported in the manuscript and Supporting Information figures. (PDF)

S1 Table Chemical proteomics. (XLSX)

S2 Table ADP-ribosylation proteomics. (XLSX)
