# Supplementary material for: Integrative proteomics reveals MSH6 to modulate PARP inhibitor sensitivity in BRCA1/2-proficient ovarian cancer
Source: PLoS One. 2026 Sep 2;21(9):e0357365. doi: 10.1371/journal.pone.0357365 (PMC13537606; doi:10.1371/journal.pone.0357365)
Supplement: Supplementary file 1 [file pone.0357365.s001.pdf]

Figure S1

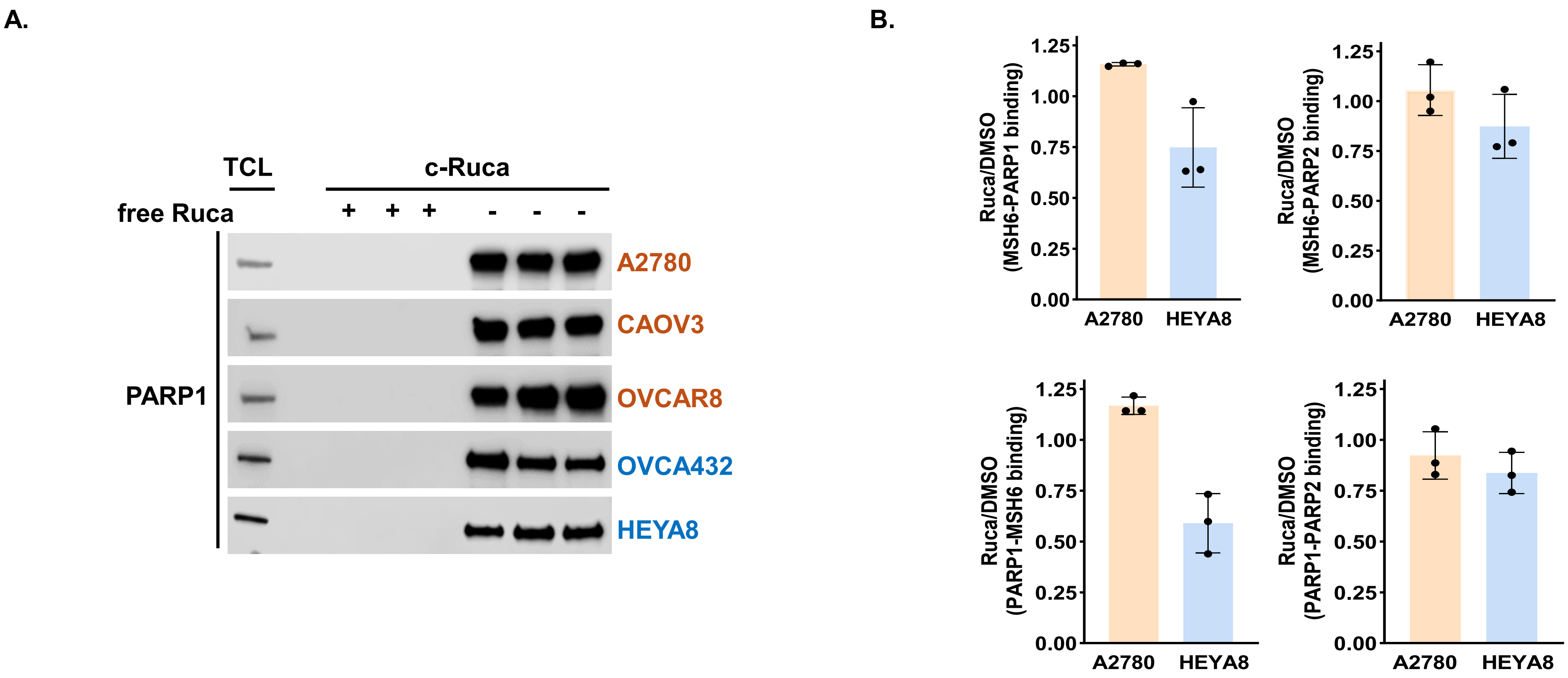


**Figure S1**. PARPi affinity matrices identify the enrichment of PARP1 in OC cells.

(A) Immunoblot of eluates from c-rucaparib-modified beads incubated with different cell lysates ±20 μM of free rucaparib. TCL, total cell lysate. Ruca: Rucaparib. (B) Quantification of PARP–MSH6 interactions from the Western blots shown in Fig. 2E. Upper panels show quantification of PARP1–MSH6 (left) and PARP2–MSH6(right) interactions assessed by MSH6 co-immunoprecipitation, expressed as the fold change in interaction following rucaparib treatment relative to DMSO. Lower panels show quantification of MSH6–PARP1 (left) and PARP2–PARP1 (right) interactions assessed by PARP1 co-immunoprecipitation, also expressed as the ratio of rucaparib-treated to DMSO-treated samples. Data are presented as mean ± SD; each dot represents an independent biological replicate.

Figure S2

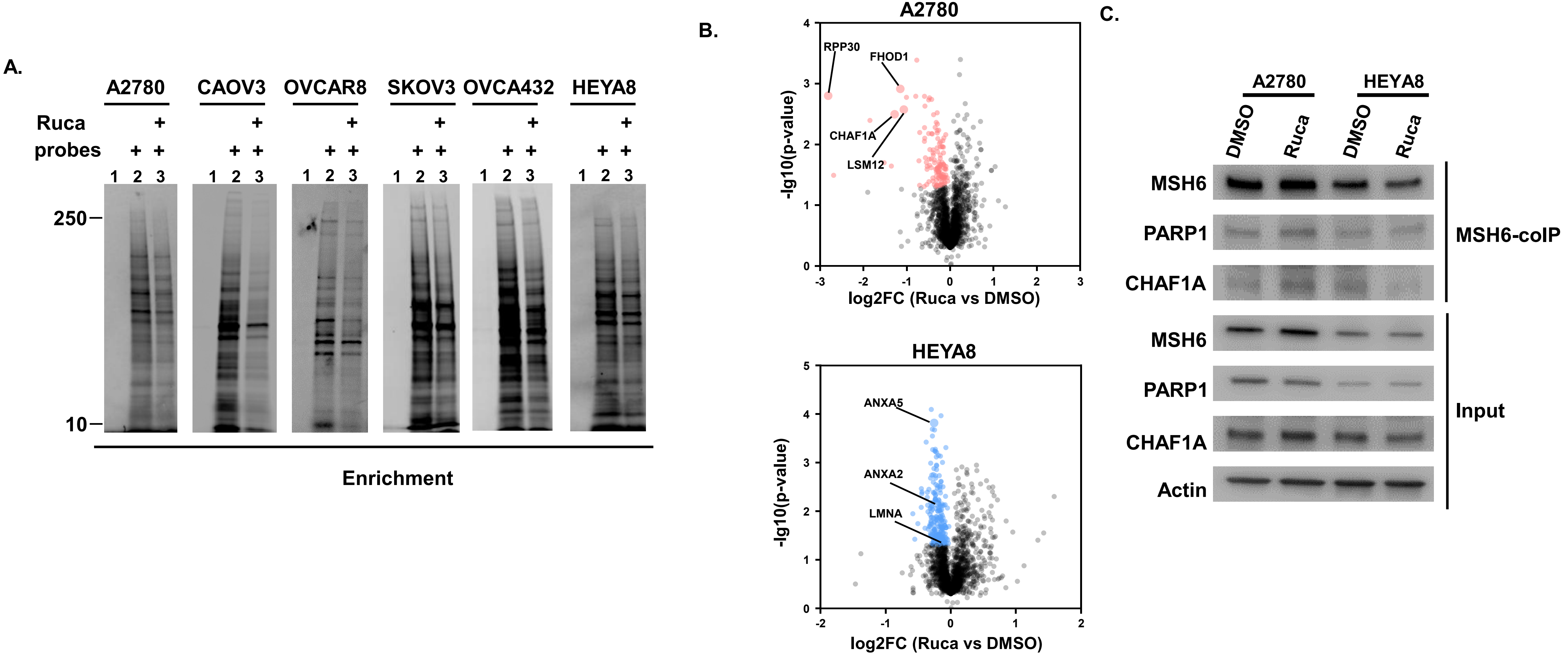


**Figure S2.** Differential ADP-ribosylation profiles and mediated signaling in response to rucaparib in BRCA-proficient OC cells. (A) Qualitative assessment of 6Yn/2YnAd (probes) labelling in different cells by in-gel fluorescence scanning. Lane 1: DMSO control; Lanes 2: A mix of 6Yn/2Yn-adenosine (0.5 mM each); lane 3: A mix of 6Yn/2Yn-adenosine with rucaparib (10 μM). Images are representative of at least two biological replicates. (B) Volcano plots indicating differential log2 responses in the level of ADP-ribosylation of endogenous proteins to 10 μM rucaparib in A2780 (upper) and HEYA8 cells (lower). (C) Western blot analysis of eluates from immunoprecipitation (IP) with IgG, MSH6 antibodies. A2780 and HEYA8 cells were treated with 10 μM rucaparib for 1 h and subjected to co-IP, followed by Western blotting with the indicated antibodies. Western blot images are representative of three biological replicates. Input: 10 μg total lysates from A2780 and HEYA8 cells.

## Figure S3

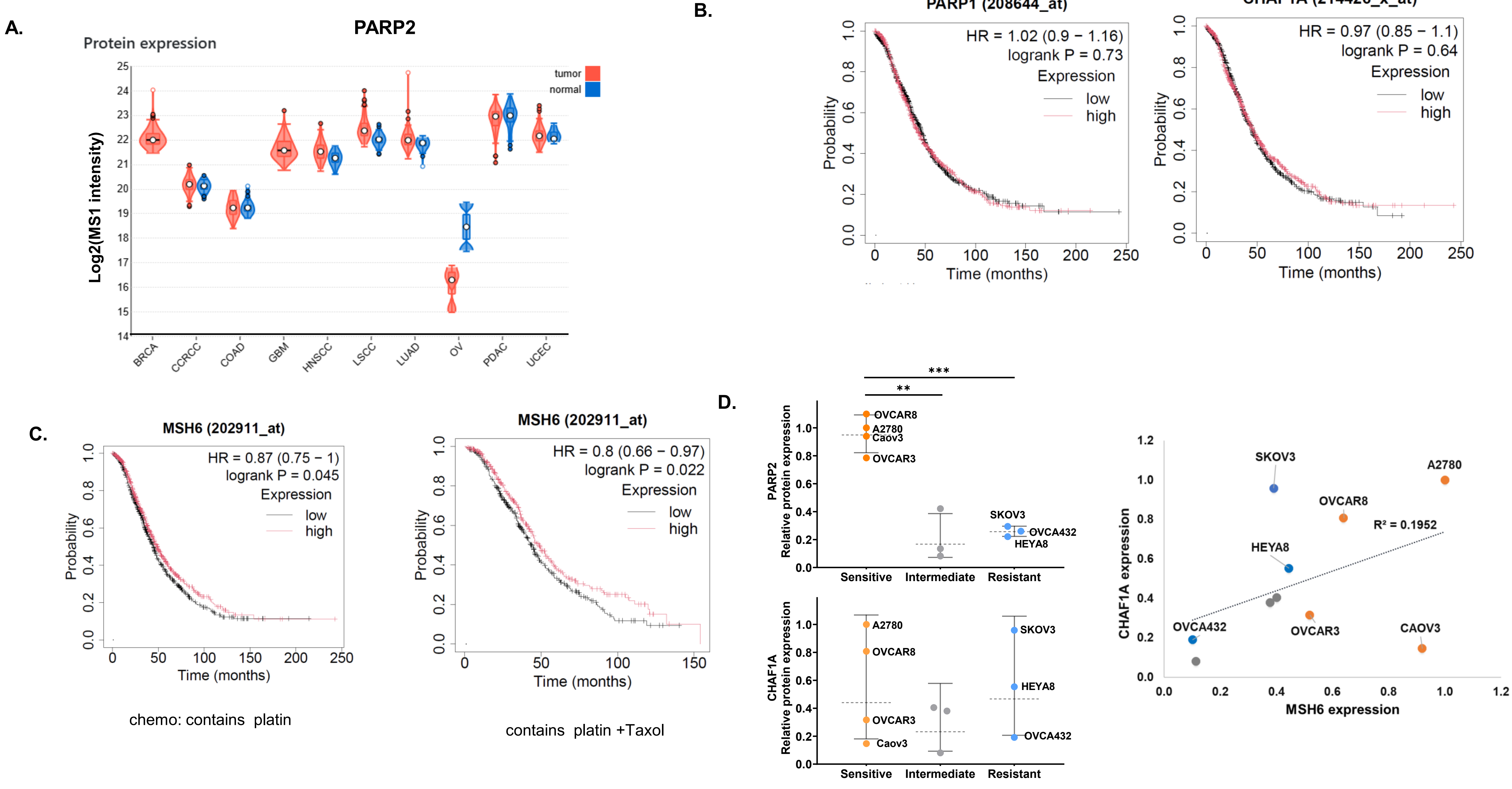
A.
PARP2
Protein expression
tumor
normal
Log2(MS1 intensity)
BRCA
CCRCC
COAD
GBM
HNSCC
LSCC
LUAD
OV
PDAC
UCEC
B.
PARP1 (208644_at)
HR = 1.02 (0.9 − 1.16)
logrank P = 0.73
Expression
low
high
Probability
Time (months)
CHAF1A (214426_x_at)
HR = 0.97 (0.85 − 1.1)
logrank P = 0.64
Expression
low
high
Probability
Time (months)
C.
MSH6 (202911_at)
HR = 0.87 (0.75 − 1)
logrank P = 0.045
Expression
low
high
Probability
Time (months)
chemo: contains platin
MSH6 (202911_at)
HR = 0.8 (0.66 − 0.97)
logrank P = 0.022
Expression
low
high
Probability
Time (months)
contains platin +Taxol
D.
***
**
PARP2
Relative protein expression
OVCAR8
A2780
Caov3
OVCAR3
SKOV3
OVCA432
HEYA8
Sensitive
Intermediate
Resistant
CHAF1A
Relative protein expression
A2780
OVCAR8
OVCAR3
Caov3
SKOV3
HEYA8
OVCA432
Sensitive
Intermediate
Resistant
SKOV3
A2780
OVCAR8
R² = 0.1952
HEYA8
OVCA432
OVCAR3
CAOV3
CHAF1A expression
MSH6 expression

**Figure S3.** Relationship between MSH6/PARP1/CHAF1A expression and overall survival.

(A) Expression level analysis of PARP2 protein using CPTAC data accessed through the LinkedOmicsKB web portal18, comparing tumor and normal tissues. BRCA: Breast Invasive Carcinoma; CCRCC: Clear Cell Renal Cell Carcinoma; COAD: Colon Adenocarcinoma; GBM: Glioblastoma; HNSCC: Head and Neck Squamous Cell Carcinoma; LSCC: Lung Squamous Cell Carcinoma; LUAD: Lung Adenocarcinoma; OV: Ovarian Serous Cystadenocarcinoma; PDAC: Pancreatic Ductal Adenocarcinoma; UCEC: Uterine Corpus Endometrial Carcinoma. (B, C) Prognostic role of gene expression in OC patients. Kaplan–Meier plot27 showing PARP1 (B, left panel), CHAF1A (B, right panel), MSH6 in the chemotherapy with platin treatment (C, left panel) or platin+ taxol (C, right panel) groups. (D) Quantification of PARP2 and CHAF1A protein expression (from Fig. 4D) shown on the left panels. Each dot represents the mean of three biological replicates per cell line. Error bars indicate the mean ± SD for each group. Protein expression values were normalized to those of the A2780. Statistical significance between groups is indicated above the plots (** $p < 0.005$, *** $P < 0.001$). The right panel indicates protein expression correlation analysis between MSH6 and CAHF1A in OC cells.

Figure S4

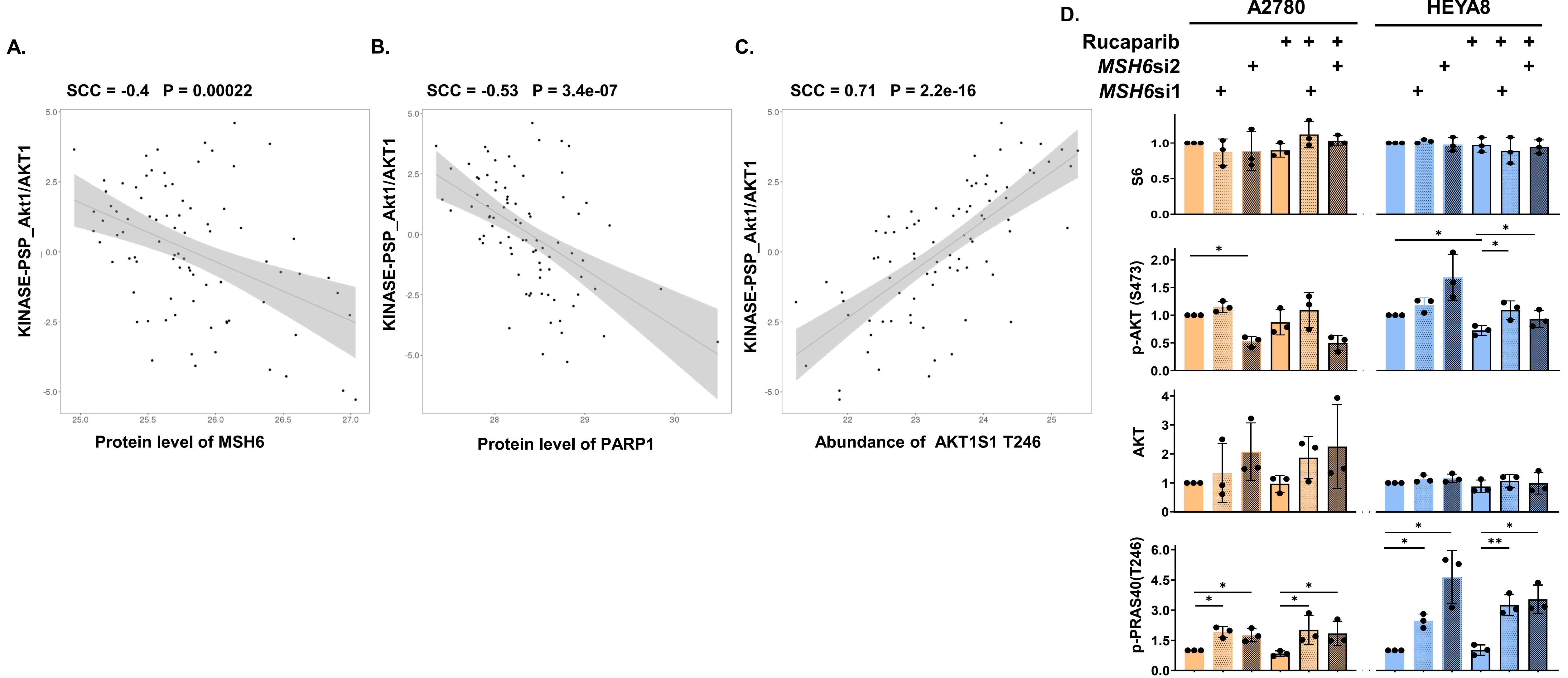


**Figure S4.** Associations of the protein abundance of MSH6 and PARP1 with pathway.
Pathway associations of the protein or phosphoprotein abundance of MSH6 (A) and PARP1 (B), as well as pPRAS40 T246 (C) through the LinkedOmicsKB web portal. (D) Quantification of S6, p-AKT(S473), AKT and p-PRAS40(T246) from Fig 4D western blot. Data are presented as mean ± SD; each dot represents a biological replicate

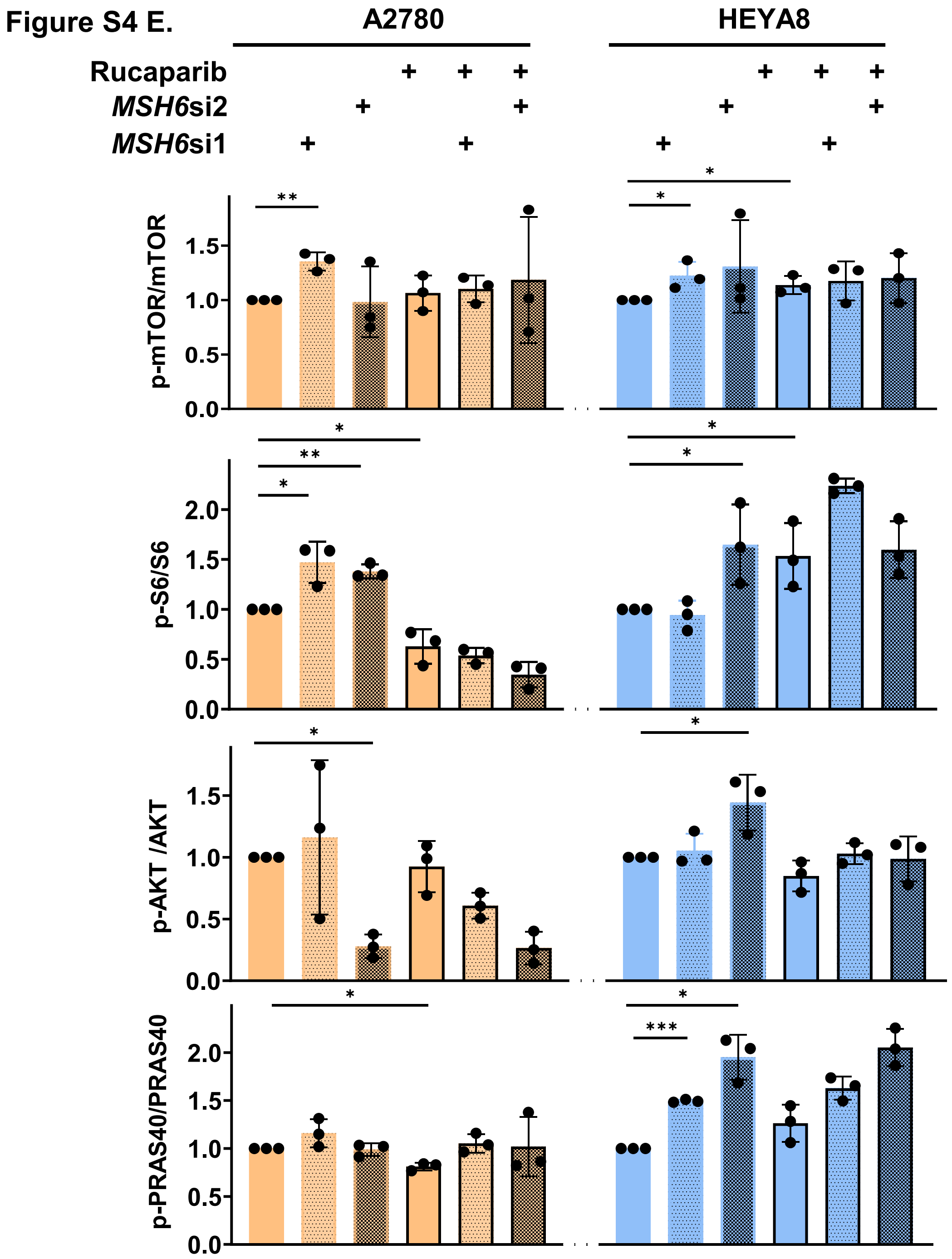


**Figure S4.**
(E) Quantification of p-mTOR / total mTOR, p-S6 / total S6, p-AKT / total AKT, and p-PRAS40 / total PRAS40 from the western blot shown in Fig 4D. Data are presented as mean ± SD, with each dot representing a biological replicate.
