# Supplementary material for: Integrative proteomics reveals MSH6 to modulate PARP inhibitor sensitivity in BRCA1/2-proficient ovarian cancer
Source: PLoS One. 2026 Sep 2;21(9):e0357365. doi: 10.1371/journal.pone.0357365 (PMC13537606; doi:10.1371/journal.pone.0357365)
Supplement: Supplementary file 2 [file pone.0357365.s002.pdf]

Supplementary Data: Uncropped Blot Images

Figure S5

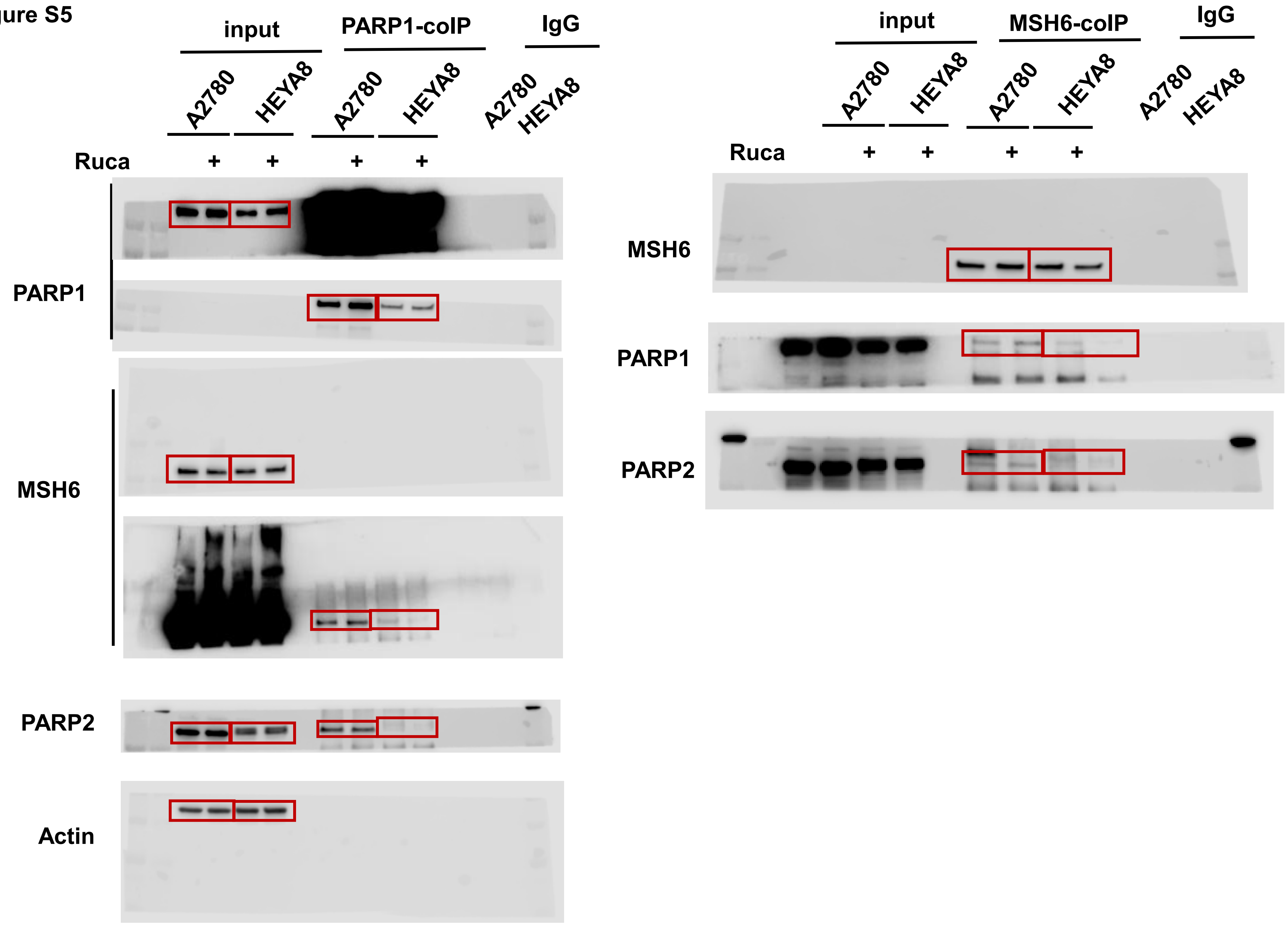

Supplementary Figure S5: Full-length, uncropped western blots corresponding to the cropped blots shown in Figure 2E. Red boxes indicate the regions used in the main figure. Input samples were run alongside the MSH6 co-IP and PARP1 co-IP in separate gels, and blots were cropped by cell line for clarity in the main figures.

Figure S6

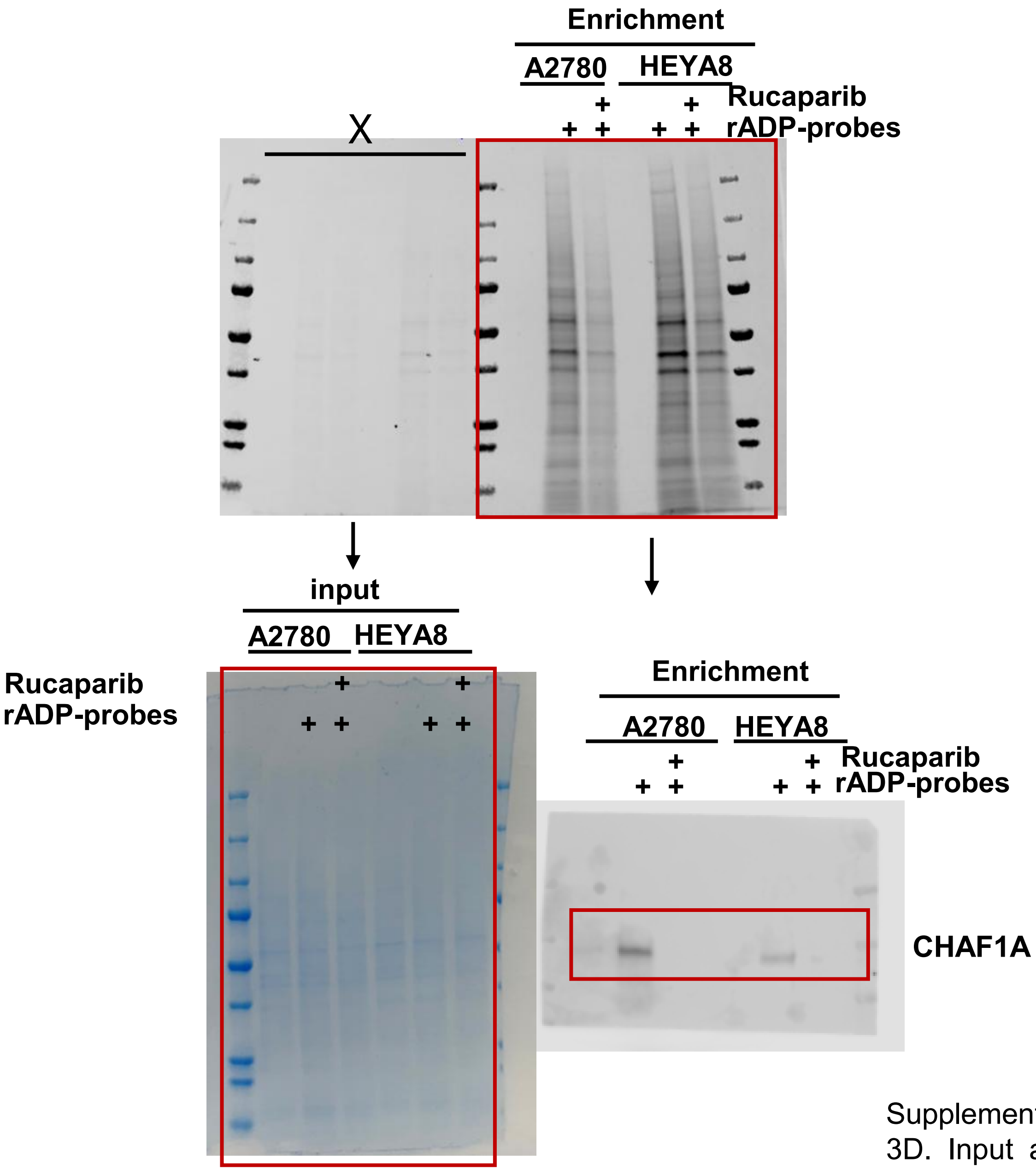

Supplementary Figure S6:Full-length, uncropped images corresponding to Figure 3D. Input and enrichment samples were resolved in the same gel, which was scanned using LI-COR prior to processing (upper panel). The input portion was stained with AcquaStain (lower left), and the enrichment portion was transferred to a membrane and probed with CHAF1A antibody (lower right). Red boxes indicate the cropped regions shown in the main figure.

Figure S7

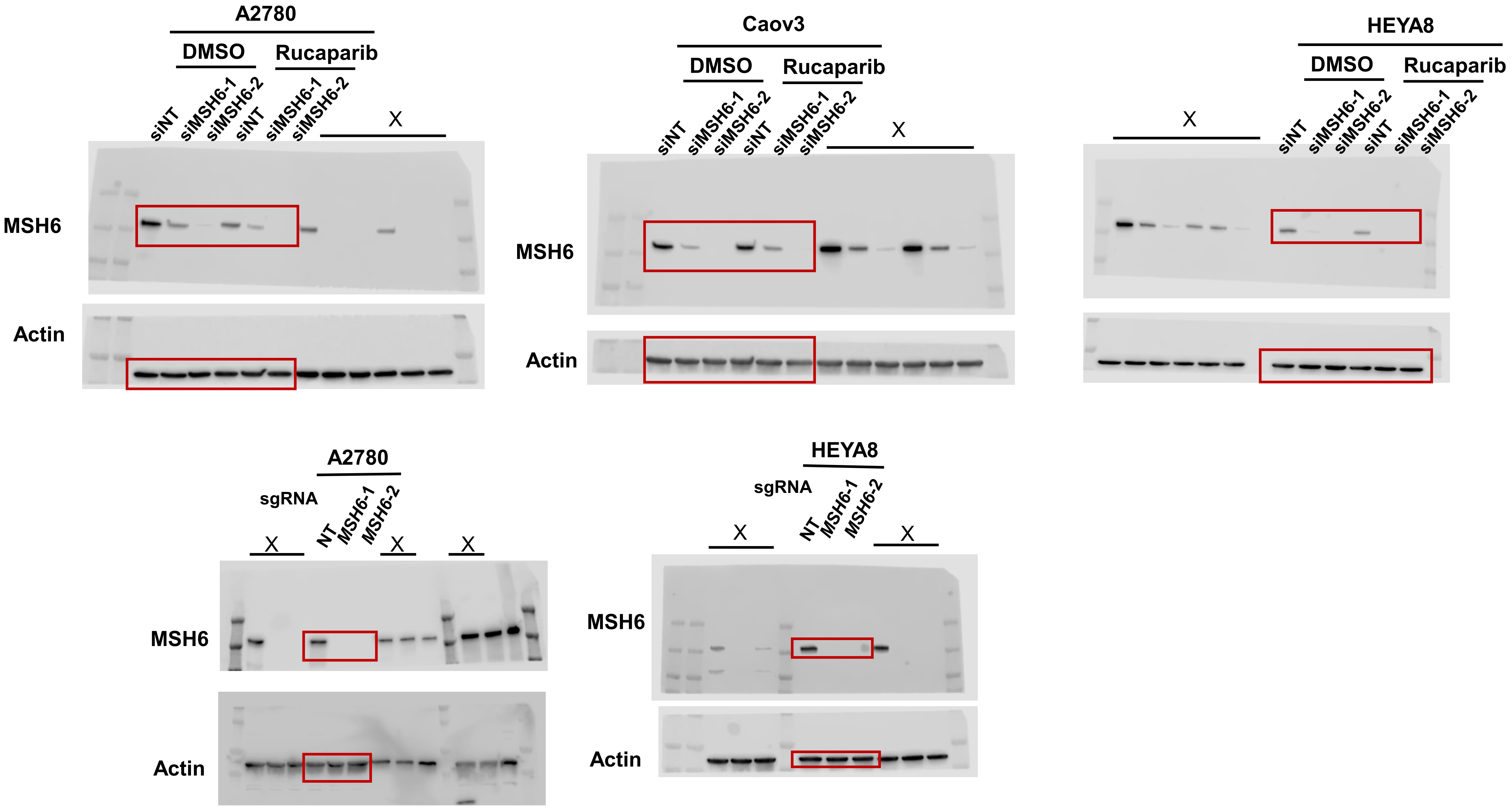

Supplementary Figure S7:Full-length, uncropped images corresponding to Main Figure 5A (upper panels) and 5B (lower panels). Blots for MSH6 and actin from each cell line were obtained from the same gel. Membranes were cut at the appropriate molecular weight regions and probed with the indicated antibodies. Red boxes indicate the regions shown in the main figure, highlighting only the samples relevant to this study.

Figure S8

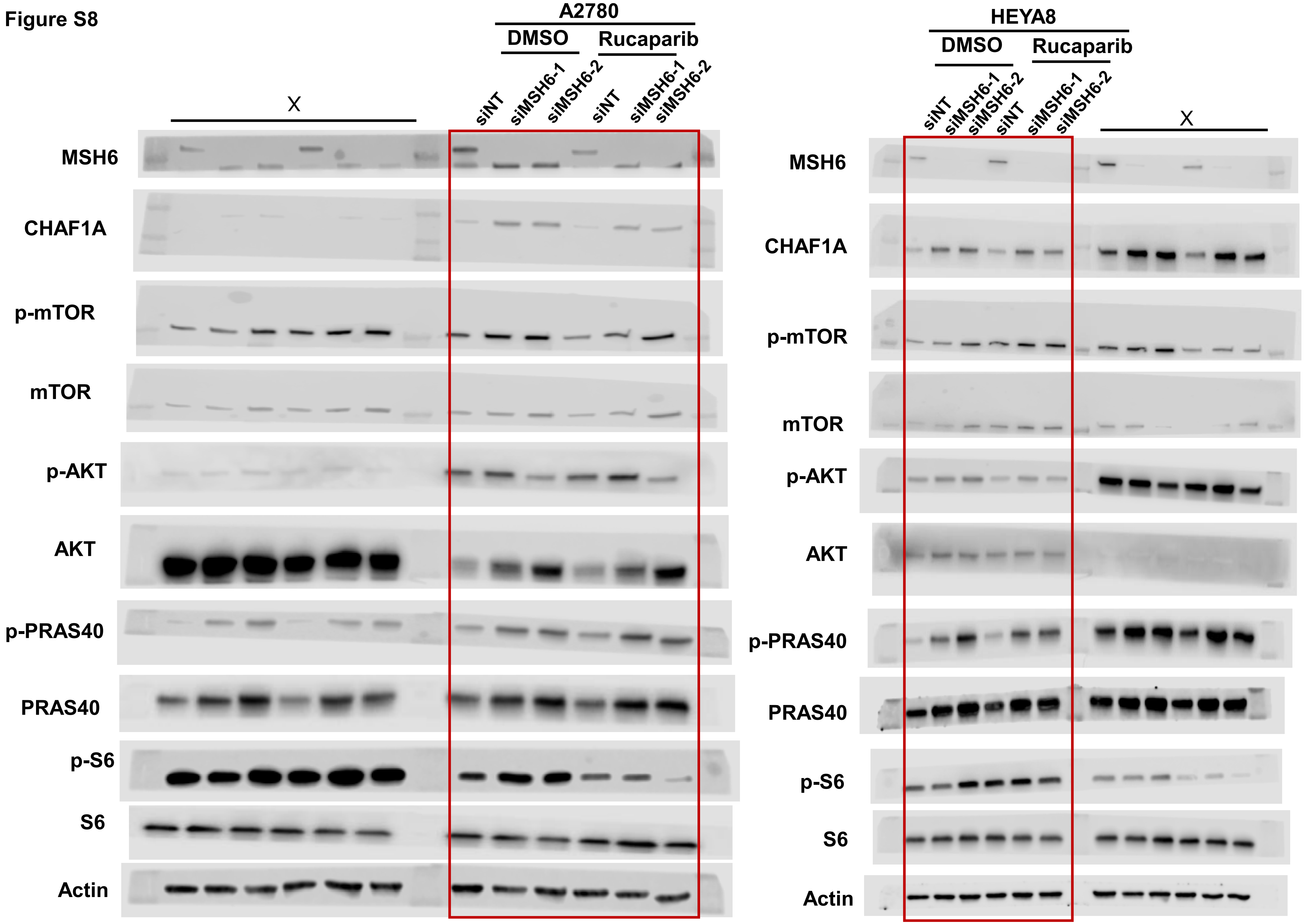

Supplementary Figure S8:Full-length, uncropped images corresponding to Main Figure 6A (left panels) and (right panels). Blots from each cell line were obtained from the same gel. Membranes were cut at the appropriate molecular weight regions and probed with the indicated antibodies. Red boxes indicate the regions shown in the main figure, highlighting only the samples relevant to this study.

Figure S9

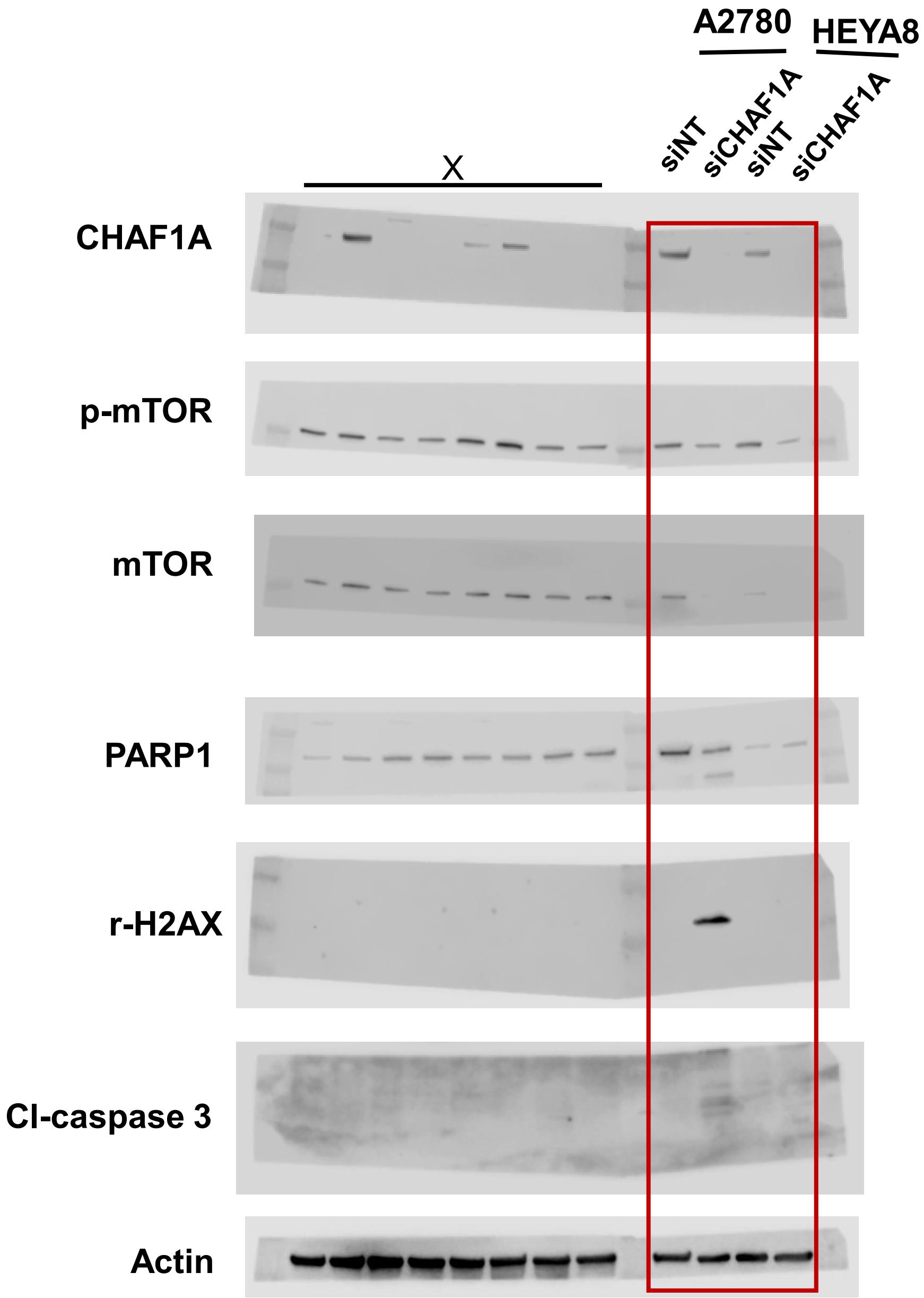

Supplementary Figure S9:Full-length, uncropped images corresponding to Main Figure 6B. Membranes were cut at the appropriate molecular weight regions and probed with the indicated antibodies. Red boxes indicate the regions shown in the main figure, highlighting only the samples relevant to this study.

Figure S10

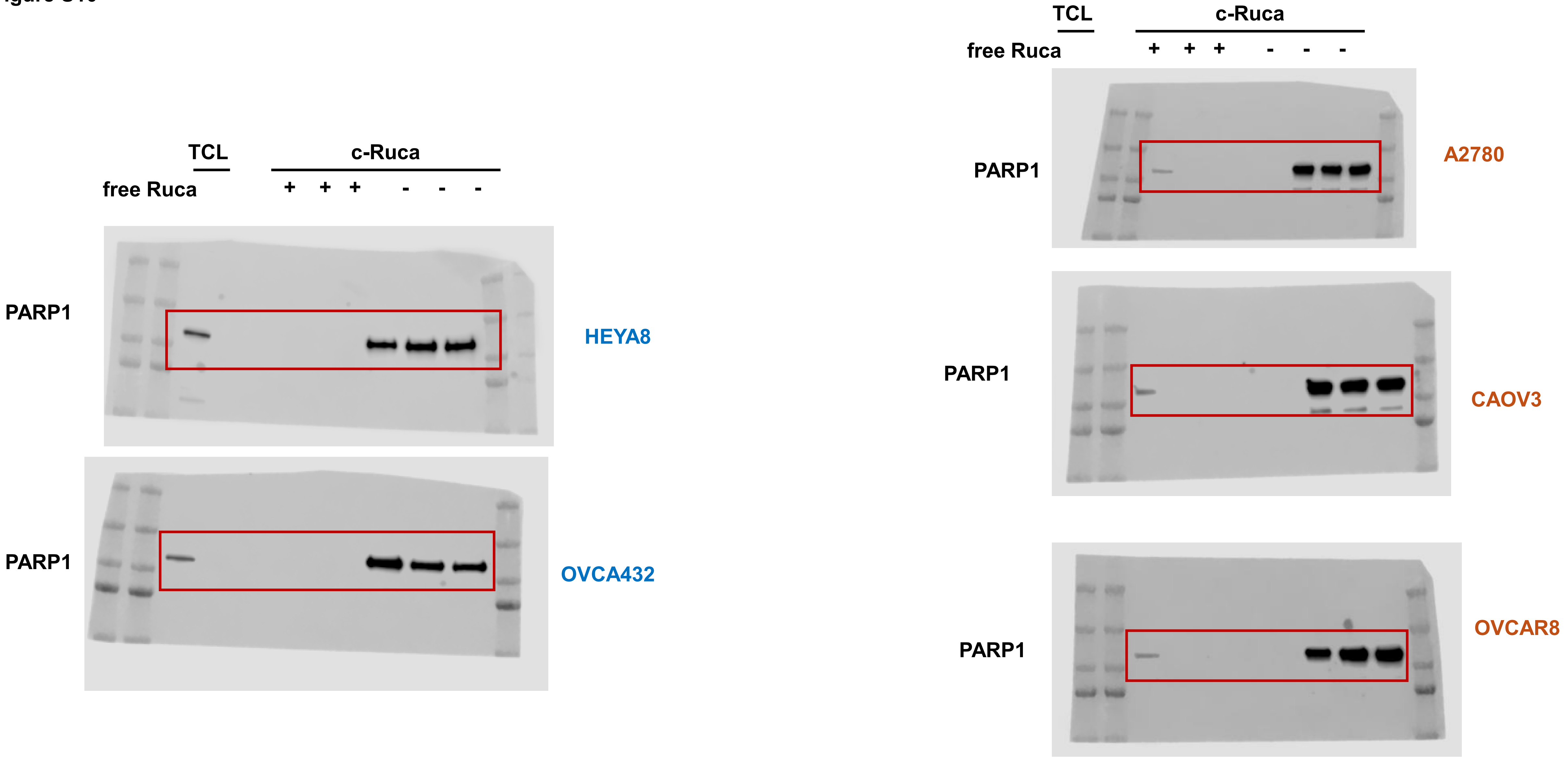

Supplementary Figure S10:Full-length, uncropped images corresponding to Main Figure S1A. Membranes were cut at the appropriate molecular weight regions and probed with the indicated antibodies.

Figure S11

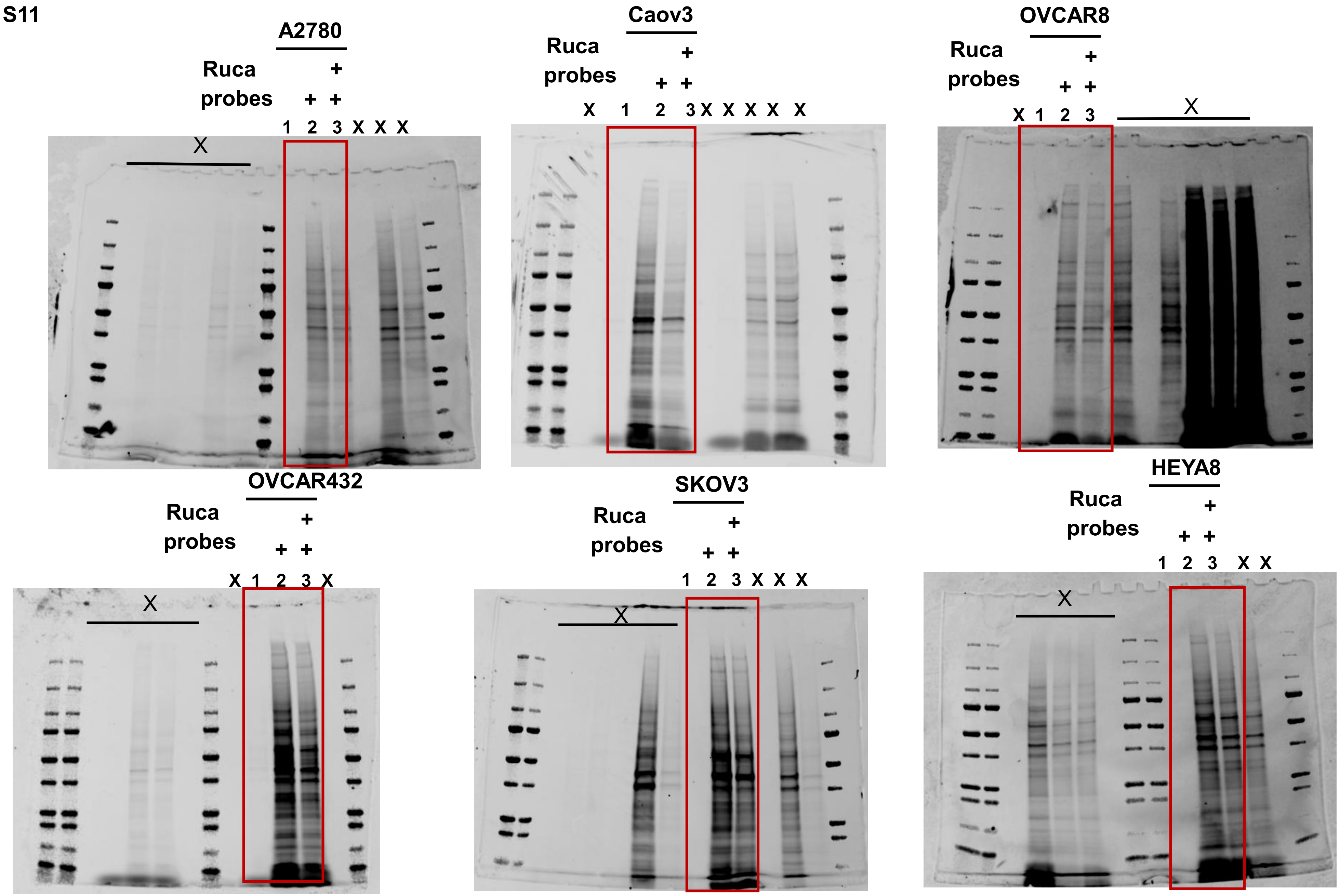

Supplementary Figure S11:Full-length, uncropped images corresponding to Figure S2A. enrichment samples were resolved in the gel, which was scanned using LI-COR prior to processing (upper panel). Red boxes indicate the cropped regions shown in the figure.

Figure S12

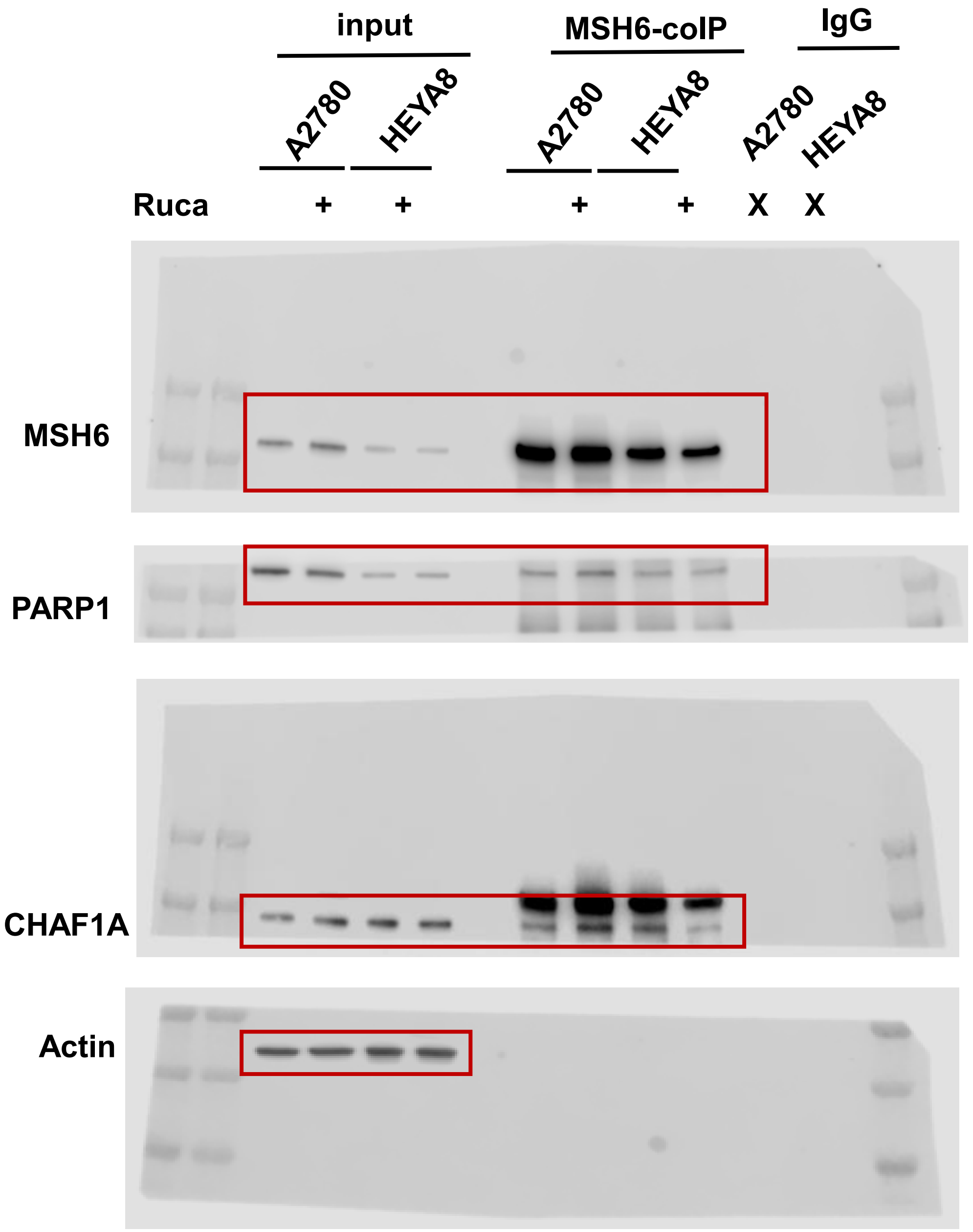

Supplementary Figure S12: Full-length, uncropped western blots corresponding to the cropped blots shown in Figure S2C. Red boxes indicate the regions used in the figure. Blots were cropped by cell line for clarity in the figures.
