# Supplementary material for: Integrative proteomics reveals MSH6 to modulate PARP inhibitor sensitivity in BRCA1/2-proficient ovarian cancer
Source: PLoS One. 2026 Sep 2;21(9):e0357365. doi: 10.1371/journal.pone.0357365 (PMC13537606; doi:10.1371/journal.pone.0357365)
Supplement: Supplementary file 3 [file pone.0357365.s006.pdf]

Response to Academic Editor

General Editorial Assessment

Comment:

The manuscript appears scientifically solid, technically rigorous, and potentially impactful, with strengths in the proteomics workflow and translational relevance. However, several concerns were raised regarding mechanistic validation, interpretation, and clinical relevance, which require additional discussion and textual revision.

Response:

We sincerely thank the Academic Editor for the positive evaluation of our work and for the constructive guidance. In response to these concerns, we substantially revised the manuscript to strengthen the discussion of mechanistic validation, clarify interpretation of the findings, and better contextualize the clinical relevance of the study. Specifically, we:

- Expanded the Discussion section regarding mechanistic implications and limitations;
- Clarified the interpretation of the proteomics findings;
- Added discussion addressing the suggested future validation experiments, including CHAF1A rescue studies, PARylation site validation, and in vivo/organoid validation approaches.

These revisions are reflected throughout the revised manuscript.

Journal Requirements

Comment 1

Please ensure that your manuscript meets PLOS ONE style requirements, including file naming and formatting.

Response:

We carefully revised the manuscript to comply with all PLOS ONE formatting and file naming requirements.

Comment 2

We noticed you have some minor occurrence of overlapping text with the following previous publication(s), which needs to be addressed:

Integrated proteomics identifies PARP inhibitor-induced prosurvival signaling changes as potential vulnerabilities in ovarian cancer - 10.1016/j.jbc.2022.102550

In your revision ensure you cite all your sources (including your own works), and quote or rephrase any duplicated text outside the methods section. Further consideration is dependent on these concerns being addressed.

Response:

We thank the editor for bringing this to our attention. We carefully reviewed our manuscript in comparison with the cited publication (DOI: 10.1016/j.jbc.2022.102550), which was already cited in the Introduction:

"In **our previous work**, we have observed that the composition of PARP1-associated protein complexes and patterns of poly-ADP-ribosylation (PARylation) may play critical roles in differentially modulating PARPi response between BRCA-deficient and BRCA-proficient OC cells [20]."

In addition, we reviewed the manuscript for potential text overlap outside the Methods section and revised the wording where appropriate. We have also verified that all relevant prior work, including our own publications, is properly cited throughout the manuscript.

Comment 3

Please amend the Funding Statement and include: "There was no additional external funding received for this study."

Response:

We updated the Funding Statement to include all funding sources and added the requested statement:
"**There was no additional external funding received for this study.**"

Comment 4

Please remove funding-related text from the manuscript.

Response:

All funding-related text has been removed from the manuscript body in accordance with PLOS ONE guidelines.

Comment 5

Please provide original uncropped and unadjusted blot/gel images.

Response:

We have provided the original uncropped and unadjusted blot/gel images as Supporting Information files, in accordance with PLOS ONE figure reporting requirements.

Comment 6

Please upload higher-quality versions of Figures 1–6.

Response:

We uploaded revised high-resolution versions of Figures 1–6 to improve image clarity and readability.

Comment 7

Please include captions for all Supporting Information files and update in-text citations accordingly.

Response:

Captions for all Supporting Information files have been added at the end of the manuscript, and all in-text citations have been updated accordingly.

Response to Reviewer #1

Main Comments

Comment 1
All experiments were performed in cell lines. This limitation should be discussed more clearly. Validation in xenograft, organoid, or patient-derived models would further support the translational relevance of the findings, if feasible.

Response:
We appreciate the reviewer's insightful comment and agree that validation in organoid, xenograft, or patient-derived models would further strengthen the translational relevance of our findings. We have therefore revised the Discussion section to explicitly acknowledge this limitation and emphasize the importance of future validation in physiologically relevant systems. Accordingly, we have added this aspect to the discussion:

"**A limitation of the present study is that it is primarily based on established ovarian cancer cell line models. Therefore, further validation experiments will also have to be performed in patient-derived organoids, xenografts, and clinical tumor specimens to establish its translational relevance and potential clinical applicability**." (Page 32)

Comment 2
The connection between MSH6 and CHAF1A is interesting, but currently appears mostly correlative. The manuscript would benefit from additional discussion of:

MSH6 regulates CHAF1A directly,

CHAF1A is required for MSH6-related PARPi

rescue experiments were considered or attempted.

Response:

We appreciate this important observation. In the revised manuscript, we toned down mechanistic interpretations regarding the relationship between MSH6 and CHAF1A and clarified that our current findings support a functional association rather than definitive direct regulation.

We further expanded the Discussion to acknowledge that the precise mechanistic relationship between MSH6 and CHAF1A remains to be established, and that rescue experiments and targeted mechanistic validation would be valuable future directions to clarify the functional hierarchy within this pathway and to define its contribution to PARPi sensitivity in BRCA-proficient ovarian cancer. Accordingly, we have added this to the discussion:

"**Although our data identify the pair-wise interactions between PARP1, MSH6 and CHAF1A and illustrate the functional effects of MSH6 on CHAF1A protein levels and PARPi sensitivity, the precise mechanistic relationship between these proteins remains to be defined. It is currently unclear whether MSH6 directly regulates CHAF1A activity or whether CHAF1A also contributes to modulating PARPi response. Further functional studies, including rescue experiments and targeted perturbation approaches, will be required to clarify these signaling mechanisms in detail.**" (Page 32)

Comment 3

The decrease in CHAF1A ADP-ribosylation is interesting, but it is not fully clear whether this reflects a specific mechanism or broader changes in PARP activity.

Response:

We agree that the observed decrease in CHAF1A ADP-ribosylation should be interpreted cautiously. Our study was designed to compare ADP-ribosylation profiles between PARPi-sensitive and -resistant cells. Following rucaparib treatment, we observed a significant reduction in CHAF1A ADP-ribosylation, and CHAF1A emerged as one of the proteins exhibiting differential ADP-ribosylation between PARPi-sensitive and -resistant cells. Considering the pronounced effects of CHAF1A silencing on cell viability, these findings suggest that CHAF1A may represent a downstream target associated with PARP1 signaling and PARPi response.

However, future studies will be important to determine the extent to which CHAF1A ADP-ribosylation contributes to PARPi sensitivity and therapeutic response. Additional mechanistic studies, including PARP1 knockout or depletion models, biochemical validation of CHAF1A ADP-ribosylation, and site-specific ADP-ribosylation analyses, will be required to further investigate the regulation of CHAF1A ADP-ribosylation. Accordingly, we have revised the Discussion to tone down the mechanistic interpretation and clarify these limitations:

"**These findings indicate that CHAF1A ADP-ribosylation is associated with PARPi response and may contribute to the differential assembly of the PARP1/MSH6/CHAF1A complex.**

However, whether CHAF1A is directly ADP-ribosylated by PARP1 or other known PARP family targets, such as PARP2 or tankyrase [37, 68], ADP-ribosylation occurs at specific functional sites or contributes directly to CHAF1A's regulatory activity requires further studies using PARP1 knockdown, site-directed mutagenesis and mapping of CHAF1A PARylation sites." (Page 30)

"**However, the functional consequences of CHAF1A ADP-ribosylation, its impact on protein interactions and PARPi response were not examined in this study and will require further investigation."** (Page 31)

Comment 4
The classification of "sensitive" and "resistant" based mainly on rucaparib Cmax may oversimplify biological differences between models.

Response:

We appreciate the reviewer's concern. However, the classification of PARPi-sensitive and -resistant models was not based solely on rucaparib Cmax. PARPi sensitivity was evaluated according to the IC50 values of all three FDA-approved PARPis across the BRCA-proficient ovarian cancer cell lines.

As described in Results section 1, we focused on rucaparib because it is the PARPi FDA-approved for recurrent ovarian cancer regardless of BRCA status. At the clinically relevant Cmax (1.94 μM), only about one-third of the tested cell lines showed >50% reduction in viability, while most exhibited limited response.

Based on these response patterns, we selected A2780, OVCAR8, and Caov3 as representative PARPi-sensitive lines and HEYA8, SKOV3, and OVCA432 as PARPi-resistant lines (Fig. 1B). OVCAR3 was excluded despite its sensitivity because of previously reported deep BRCA2 deletions, which could confound interpretation of BRCA-proficient PARPi responses.

We have now rewritten this sentence to reflect the sensitivity/resistance to the other PARPis more clearly, now reads (changes in bold):

"Based on their IC50 values, relative to rucaparib's maximal plasma concentration (1.94 mM), **and consistency across the other PARPis olaparib and niraparib,** cell lines were classified as sensitive or resistant (Fig 1B)." (Page 14)

Comment 5
Since MSH6 is an important mismatch repair protein, the Discussion should more fully address mismatch repair deficiency, microsatellite instability, and their possible relationship with PARPi response.

Response:

We thank the reviewer for this important suggestion. We agree that MSH6 is a core component of the DNA mismatch repair (MMR) pathway and is frequently discussed in

the context of mismatch repair deficiency and microsatellite instability (MSI), which have been associated with genome instability and therapeutic responses in cancer.

However, MSI-high ovarian cancers are relatively uncommon, and the majority of ovarian cancers, including the models used in our study, are microsatellite stable (MSS). Therefore, the biological context of our study differs from that of MSI-associated malignancies in which MSH6 dysfunction has been more extensively investigated.

Previous studies investigating MSH6 in the context of PARP inhibitor response have largely focused on MMR deficiency, MSI, or genetic alterations of MMR pathway components. In contrast, the ovarian cancer models examined in the present study are predominantly microsatellite stable (MSS) and BRCA-proficient. Therefore, our findings relate to differential MSH6 protein expression and protein complex formation rather than classical MMR deficiency. To our knowledge, the association between MSH6 protein expression and PARP inhibitor sensitivity in BRCA-proficient ovarian cancer has not been systematically investigated.

We have revised the Discussion to explicitly acknowledge the canonical role of MSH6 in MMR and its established relationship with MSI. However, our experimental findings suggest that MSH6 may also have functions beyond its traditional role in mismatch repair. Notably, depletion of MSH6 did not significantly affect cell viability, indicating that the observed effects are unlikely to be explained solely by loss of canonical MMR activity. Instead, our data supports a model in which MSH6 contributes to PARP inhibitor response through altered protein interactions, including changes in PARP1-associated complexes and downstream signaling pathways. Nevertheless, the precise mechanisms linking MSH6 expression to PARP inhibitor sensitivity remain to be fully elucidated and warrant further investigation. These revisions have been incorporated into the Discussion:

"**MSH6 has been extensively studied in the context of MMR deficiency and microsatellite instability (MSI) [57-60]. However, MSI-high tumors are relatively uncommon in ovarian cancer, occurring in approximately 1–3% of serous cases [61-65]. Although MSI status was not directly assessed in the present study, previous reports have classified SKOV3 as MSI-H, whereas the remaining cell lines used in this study are generally considered microsatellite stable (MSS) [66, 67]. Therefore, the differential MSH6 expression and PARP1/MSH6 complex formation observed in our models are unlikely to be explained solely by classical MMR deficiency. Instead, these findings support the possibility that MSH6 may have additional functions beyond canonical mismatch repair, potentially through PARP1-associated signaling pathways that influence PARPi response. The relationship between MSH6-associated signaling, MSI phenotypes, and PARPi sensitivity warrants further investigation.**" (Page 29-30)

Comment 6
More detail on statistical analysis would improve clarity.

Response:

Additional details regarding statistical analyses have now been included in the Methods section:

“Data was obtained from at least three independent experiments and shown as the mean ± SD. Data was analyzed using Microsoft Excel and GraphPad Prism 10.0. Statistical analyses were performed using Student’s t test **(paired or unpaired, as appropriate)** and synergy was determined by the Bliss independence model[35]. **P value of <0.05 was considered statistically significant.** All statistical details are included in the figure legends. All MaxQuant data were first filtered for peptides with PEP score < 0.05. Furthermore, reverse and contaminant peptides and peptides with no intensity were excluded. Data were then normalized using IRON[41].” (Page 13)

Minor Comments

1. Some figure legends are too long and could be simplified.

2. In several places, associative wording may be more appropriate than causal wording.

3. The Discussion would benefit from additional comparison with previous studies on PARP1, mismatch repair, and replication fork stability.

4. Minor grammar and formatting issues should be corrected.

5. Abbreviations should be used consistently throughout the manuscript.

Response:
We thank the reviewer for these suggestions. Figure legends were revised for clarity and conciseness, causal wording was modified where appropriate, additional literature comparisons were incorporated into the Discussion, and the manuscript was carefully edited for grammar, formatting consistency, and standardized abbreviation usage throughout.

Questions for the Authors

Question 1
Were rescue experiments with CHAF1A restoration considered or performed?

Response:
We agree that such experiments would provide important mechanistic validation and would have liked to engage in these experiments**:**

“A limitation of the present study is that it is primarily based on established ovarian cancer cell line models. Therefore, further validation experiments will also have to be performed in patient-derived organoids, xenografts, and clinical tumor specimens to establish its translational relevance and potential clinical applicability.” (Page 32)

Question 2

Are there public datasets linking MSH6 expression with PARPi response in BRCA-proficient ovarian cancer?

Response:
To the best of our knowledge, as PARPis have been approved for ovarian cancer only for a few years so far and not as first-line therapy, there are currently no large, dedicated public clinical datasets that directly stratify PARPi response by MSH6 expression specifically in BRCA-proficient ovarian cancer cohorts.

Question 3
Have the authors considered validation in organoid or in vivo models?

Response:
Yes. We agree that validation in organoid and in vivo systems would substantially strengthen translational relevance. These studies are planned as part of future work and are now acknowledged in the Discussion section:

"A limitation of the present study is that it is primarily based on established ovarian cancer cell line models. Therefore, further validation experiments will also have to be performed in patient-derived organoids, xenografts, and clinical tumor specimens to establish its translational relevance and potential clinical applicability." (Page 32)

Question 4
Did the authors examine HR-related markers such as RAD51 foci after MSH6 depletion?

Response:
RAD51 foci formation was not evaluated in the current study. We agree that assessment of homologous recombination-related markers would provide additional mechanistic insight into the relationship between MSH6 depletion and PARPi response. MSH6 has been implicated in multiple DNA damage repair pathways, including mismatch repair and double-strand break repair [57-60]., and has been reported to interact with components of both homologous recombination [58] and non-homologous end joining pathways (Shahi, Ankita et al. "Mismatch-repair protein MSH6 is associated with Ku70 and regulates DNA double-strand break repair." Nucleic acids research vol. 39,6 (2011): 2130-43. doi:10.1093/nar/gkq1095). Therefore, whether the rescue of rucaparib-induced cell death following MSH6 knockdown is associated with altered RAD51 recruitment remains unclear.

While RAD51 foci analysis is beyond the scope of the current study, we agree that further investigation of the role of MSH6 in DNA damage repair pathway choice and PARP inhibitor response would be of considerable interest and represents an important direction for future work.

Question 5
How did the authors distinguish specific PARylation effects from global PARP inhibition

effects?

Response:

We thank the reviewer for this important comment. We agree that our study does not definitively distinguish specific PARP1-mediated ADP-ribosylation events from broader effects resulting from global PARP inhibition. Our experimental strategy was designed to identify ADP-ribosylation changes associated with PARP inhibitor sensitivity by comparing PARPi-sensitive and -resistant OC cell lines under both control and rucaparib-treated conditions.

To reduce the likelihood of identifying general PARP inhibition effects, we applied multiple filtering criteria. First, we identified proteins differentially ADP-ribosylated between PARPi-sensitive and -resistant cells under basal conditions. We then focused on proteins whose ADP-ribosylation levels were significantly reduced following rucaparib treatment, particularly in PARPi-sensitive cells. This comparative approach allowed us to prioritize ADP-ribosylation events that correlate with PARPi sensitivity rather than global changes induced by PARP inhibition alone.

Nevertheless, we acknowledge that the observed changes may still reflect indirect consequences of PARP inhibition or contributions from other PARP family members, such as PARP2 or tankyrase, which we and others have reported as PARP family off-targets before **[37,68]**. Therefore, our data identify CHAF1A and other candidates as PARPi sensitivity-associated ADP-ribosylation targets, but do not necessarily establish direct PARP1-dependent modification. Additional studies, such as PARP1 knockout or depletion models, in vitro ADP-ribosylation assays, and site-specific validation experiments, will be required to distinguish direct PARP1-mediated regulation from broader PARP inhibition effects as we elaborate in the revised manuscript:

"**However, whether CHAF1A is directly ADP-ribosylated by PARP1 or other known PARP family targets, such as PARP2 or tankyrase [37, 68], ADP-ribosylation occurs at specific functional sites or contributes directly to CHAF1A's regulatory activity requires further studies using PARP1 knockdown, site-directed mutagenesis and mapping of CHAF1A PARylation sites.**" (Page 30)

Response to Reviewer #2

We sincerely thank the reviewer for the positive evaluation of our study and for the constructive and insightful comments. We appreciate the recognition of the technical rigor of our multi-platform proteomic workflow and the potential relevance of our findings. We have carefully revised the manuscript to address all concerns and provide clarifications as detailed below.

Comment 1

In the survival analysis (Fig. 4), patients receiving platinum and taxane-based chemotherapy were analyzed. How about patients receiving PARP inhibitors? Is MSH6 related to clinical outcomes of patients receiving PARPi?

Response:

We thank the reviewer for this important question.
Publicly available datasets with sufficiently powered PARPi-treated ovarian cancer cohorts and matched multi-omics survival data are limited as these are relatively new treatment options. In the current study, we therefore relied on CPTAC and available ovarian cancer cohorts primarily treated with standard platinum- and taxane-based chemotherapy to evaluate associations between MSH6 expression and clinical outcome.
We now clarify this limitation in the revised Discussion and emphasize that while MSH6 correlates with mTOR signaling activity and PARPi response in cells, its predictive value for clinical PARPi response remains to be validated in prospective PARPi-treated patient cohorts:

"**Although our data identify the interactions of PARP1, MSH6 and CHAF1A and illustrate the functional effects of MSH6 on CHAF1A protein levels and PARPi sensitivity, the precise mechanistic relationship between these proteins remains to be defined. It is currently unclear whether MSH6 directly regulates CHAF1A activity or whether CHAF1A also contributes to modulating PARPi response. Further functional studies, including rescue experiments and targeted perturbation approaches, will be required to clarify these signaling mechanisms in detail."** (Page 32)

Comment 2
The proposed role of CHAF1A PARylation is largely associative. Direct evidence is lacking demonstrating functional dependence on CHAF1A PARylation sites.

Response:
We agree with the reviewer that although the method we use, which has been developed also successfully applied by others **[33]**, our data do not provide direct functional validation of specific CHAF1A PARylation sites. The current findings are based on proteomic detection of differential ADP-ribosylation and interaction dynamics rather than site-directed mutagenesis or biochemical validation.
Accordingly, we have revised the manuscript to explicitly state that the observed CHAF1A PARylation changes are correlative and may reflect broader PARP1-mediated signaling events. We further emphasize that future studies using site-specific mutagenesis and functional assays will be required to determine whether CHAF1A PARylation directly regulates its role in PARPi sensitivity:

"**These findings indicate that CHAF1A ADP-ribosylation is associated with PARPi response and may contribute to the differential assembly of the PARP1/MSH6/CHAF1A complex.**

However, whether CHAF1A is directly ADP-ribosylated by PARP1 or other known PARP family targets, such as PARP2 or tankyrase [37, 68], ADP-ribosylation occurs at specific functional sites or contributes directly to CHAF1A's regulatory activity requires further studies using PARP1 knockdown, site-directed mutagenesis and mapping of CHAF1A PARylation sites." (Page 30)

Comment 3

The translational relevance is promising but a bit overstated. Claims regarding biomarker potential for MSH6 and PARP1 should be tempered because patient survival analyses are modest and largely indirect.

Response:
We appreciate this important critique and have tempered the language regarding biomarker potential. We now clarified that:
Current clinical evidence is indirect and exploratory
Survival associations are based on non-PARPi-treated cohorts
The proposed MSH6–PARP1 axis requires prospective validation in PARPi-treated clinical samples
We have revised the Discussion sections to avoid overstatement and to more accurately reflect the preclinical nature of the findings:

“**Although our data identify the interactions of PARP1, MSH6 and CHAF1A and illustrate the functional effects of MSH6 on CHAF1A protein levels and PARPi sensitivity, the precise mechanistic relationship between these proteins remains to be defined. It is currently unclear whether MSH6 directly regulates CHAF1A activity or whether CHAF1A also contributes to modulating PARPi response. Further functional studies, including rescue experiments and targeted perturbation approaches, will be required to clarify these signaling mechanisms in detail.”** (Page 32)

Comment 4
Overall, this is a strong and potentially impactful study, but additional mechanistic validation and more cautious interpretation are needed before publication in a high-impact setting.

Response:
We sincerely thank the reviewer for this balanced assessment. In response, we have revised the manuscript by toning down mechanistic claims regarding CHAF1A PARylation and MSH6-CHAF1A regulation. We also acknowledge the need for in vivo validation, PARylation site mapping, and functional rescue experiments:

“However, whether CHAF1A is directly ADP-ribosylated by PARP1 or other known PARP family targets, such as PARP2 or tankyrase [37, 68], ADP-ribosylation occurs at specific functional sites or contributes directly to CHAF1A’s regulatory activity requires further studies using PARP1 knockdown, site-directed mutagenesis and mapping of CHAF1A PARylation sites.” (Page 30)

“Although our data identify the interactions of PARP1, MSH6 and CHAF1A and illustrate the functional effects of MSH6 on CHAF1A protein levels and PARPi sensitivity, the precise mechanistic relationship between these proteins remains to be defined. It is currently unclear whether MSH6 directly regulates CHAF1A activity or whether CHAF1A also contributes to modulating PARPi response. Further functional studies, including rescue experiments and targeted perturbation approaches, will be required to clarify these signaling mechanisms in detail. A limitation of the present study is that it is primarily based on

established ovarian cancer cell line models. Therefore, further validation experiments will also have to be performed in patient-derived organoids, xenografts, and clinical tumor specimens to establish its translational relevance and potential clinical applicability."(Page 32)

Minor Comment
Line 296–297: incorrect statement regarding FDA approval of PARP inhibitors

Response:
We thank the reviewer for identifying this error. We have corrected the statement. The corrected sentence now reads:

"Considering that rucaparib was FDA-approved as maintenance therapy for recurrent OC regardless of BRCA mutation status, we focused our analysis primarily on this PARPi."
