# Supplementary material for: Integrative proteomics reveals MSH6 to modulate PARP inhibitor sensitivity in BRCA1/2-proficient ovarian cancer
Source: PLoS One. 2026 Sep 2;21(9):e0357365. doi: 10.1371/journal.pone.0357365 (PMC13537606; doi:10.1371/journal.pone.0357365)
Supplement: Supplementary file 4 [file pone.0357365.s007.pdf]

Response to Reviewer #1

We sincerely thank the reviewer for the thoughtful and constructive comments. We appreciate the positive assessment of our study and have revised the manuscript accordingly. Our point-by-point responses are provided below.

Comment 1. The mechanistic transition between MSH6 loss and mTOR activation needs clearer bridging. The text posits that CHAF1A acts downstream of MSH6 to regulate mTOR, but the exact mechanism remains speculative. Clarify this boundary in the Discussion.

Response:

We thank the reviewer for this important comment. We agree that the mechanistic relationship between MSH6 loss, CHAF1A regulation, and mTOR activation remains incompletely defined. We have revised the Discussion to clarify this mechanistic boundary. The revised text now reads:

"These findings link MSH6, probably through modulation of CHAF1A–mTOR signaling, to PARPi resistance in BRCA-proficient OC. **However, whether and how CHAF1A is directly regulated by MSH6 or serves as a critical mediator connecting MSH6 loss to mTOR activation remains to be determined.**" (Page 31)

Comment 2. Line 74–75: Clarify the criteria for "BRCA-proficient" upfront (e.g., absence of somatic/germline pathogenic mutations and functional HR proficiency where validated).

Response:

We thank the reviewer for this suggestion. We have clarified the criteria for "BRCA-proficient" in the Introduction. The revised text now reads:

"In this study, we investigated the proteomic landscape associated with PARPi sensitivity in a diverse panel of BRCA-proficient OC cell lines, defined here by the absence of BRCA1/2 mutations or genomic deletions. **Most cell lines used in this study have previously been reported to exhibit** HR proficiency [21-24]." (Page 4)

Comment 3. Line 88: Standardize cell line naming: ensure consistency between HEAYA8 (Line 85), HEAY8 (Line 353), and HEYA8 (used elsewhere). Standardize to HEYA8 across all sections

Response:

We thank the reviewer for identifying this inconsistency. The manuscript has been carefully reviewed, and all instances have been corrected to the standardized cell line name, HEYA8, throughout the manuscript.

Comment 4.

Results

Selecting A2780, OVCAR8, and Caov3 as sensitive lines versus HEYA8, SKOV3, and OVCA432 as resistant lines is well-justified.

A2780 is widely acknowledged as an endometrioid/undifferentiated line rather than classic high-grade serous ovarian carcinoma (HGSOC). While acceptable for general BRCA-proficient OC modeling, add a brief parenthetical note or statement in the Discussion clarifying cell line histotypes.

Response:

We appreciate this important comment. A brief statement has been added to the Discussion acknowledging that A2780 is an endometrioid/undifferentiated ovarian cancer cell line. The revised text now reads:

"To further investigate the mechanisms underlying PARPi sensitivity and resistance, we employed chemical proteomics and ADP-ribosylation proteomics to examine PARP1 protein complexes and PARylation profiles in PARPi-sensitive and -resistant BRCA-proficient OC cells**, including the endometrioid OC cell line A2780**." (Page 28)

Comment 5. Highlight the novelty of MSH6 enrichment over MSH2, which suggests an unconventional MMR-independent assembly.

Response:

We thank the reviewer for this insightful comment. We agree that the selective enrichment of MSH6 over MSH2 represents an important observation of our study. We have revised the Discussion to further emphasize the potential significance of this finding. The revised text now reads:

"Although PARP1 and MSH6 have been previously reported to interact during mismatch repair (MMR)[55, 56], particularly during the recognition and signaling of DNA mismatches, our chemical proteomics data demonstrated selective enrichment of PARP1/MSH6 complexes in PARPi-sensitive cells with no apparent enrichment of MSH2. This finding suggests **an unconventional,** potentially MMR-independent role for MSH6 in modulating PARPi sensitivity." (Page 29)

Comment 6.

Discussion

Consider adding a concise summary paragraph at the start of the Discussion that explicitly contrasts sensitive versus resistant states:

Sensitive cells: High MSH6 → PARP1–MSH6–CHAF1A complex intact → PARP1 inhibition reduces CHAF1A PARylation → suppression of mTOR signaling → cell death/synthetic lethality.

Resistant cells: Low MSH6 / complex dissociation → elevated CHAF1A → compensatory mTORC1 pathway activation → survival (targetable with mTOR/S6K inhibitors).

Response:

We appreciate this suggestion. We have added a concise summary paragraph at the beginning of the Discussion. The new paragraph summarizes the differences in MSH6-associated PARP1 complex formation, CHAF1A ADP-ribosylation and expression, mTOR signaling, and cellular response to rucaparib, and highlights the potential therapeutic relevance of mTOR pathway inhibition in PARPi-resistant cells.

"**Overall, our findings support a model in which PARPi-sensitive and PARPi-resistant BRCA-proficient OC cells exhibit distinct PARP1-associated protein complexes and downstream signaling. PARPi-sensitive compared to PARPi-resistant cells preferentially featured a PARP1-MSH6-CHAF1A complex and higher CHAF1A ADP-ribosylation, reduced by PARPi treatment, which prevented cells to launch PARPi-adaptive mTOR survival signaling. In contrast, PARPi-resistant cells showed compensatory activation of mTOR signaling following PARPi treatment and enhancement of rucaparib effects by pharmacological inhibition of the mTOR pathway. These findings suggest that compensatory mTOR signaling contributes to PARPi resistance and that mTOR pathway inhibition may be a potential combination strategy for PARPi-resistant cells.**" (Page 28)

Comment 7. Study Limitations: Expand slightly on the limitations, specifically noting the need for future in vivo PDX/allograft validation and broader clinical cohort verification for MSH6/PARP1 co-expression as a predictive biomarker.

Response:

We thank the reviewer for this valuable suggestion. We have expanded the Limitations section to further acknowledge the need for in vivo validation and evaluation of MSH6/PARP1 co-expression as a predictive biomarker for PARPi response.

The revised text now reads:

"A limitation of the present study is that it is primarily based on established ovarian cancer cell line models. Therefore, further validation experiments will also have to be performed in patient-derived xenografts **(PDX) and organoids (PDO)**, as well as clinical tumor specimens to establish its translational relevance and potential clinical applicability. **In particular, evaluation of MSH6/PARP1 co-expression in larger clinical cohorts will be required to determine its potential utility as a predictive biomarker for PARPi response**." (Page 32)
