# Supplementary material for: Integrative proteomics reveals MSH6 to modulate PARP inhibitor sensitivity in BRCA1/2-proficient ovarian cancer
Source: PLoS One. 2026 Sep 2;21(9):e0357365. doi: 10.1371/journal.pone.0357365 (PMC13537606; doi:10.1371/journal.pone.0357365)
Supplement: Peer review history

# Peer review: Decision Letter 0

**DOI:** 10.1371/journal.pone.0357365.r001

**Authors:** Giovanni Nassa

13 May 2026

Dear Dr. Deng,

Thank you for submitting your manuscript to PLOS ONE. After careful consideration, we feel that it has merit but does not fully meet PLOS ONE’s publication criteria as it currently stands. Therefore, we invite you to submit a revised version of the manuscript that addresses the points raised during the review process.

==============================

Based on the reviewers’ comments, the manuscript appears scientifically solid, technically rigorous, and potentially impactful, with strengths in the proteomics workflow and translational relevance. However, both reviewers raise several substantive concerns regarding mechanistic validation, interpretation, and clinical relevance that require additional discussion and likely textual revision rather than extensive new experimentation. Although some reviewers suggest additional mechanistic experiments (e.g., CHAF1A rescue, PARylation site validation, in vivo/organoid validation), these are framed more as desirable strengthening experiments than essential requirements for supporting the current conclusions.

==============================

Please submit your revised manuscript by Jun 27 2026 11:59PM. If you will need more time than this to complete your revisions, please reply to this message or contact the journal office at plosone@plos.org. When you're ready to submit your revision, log on to https://www.editorialmanager.com/pone/ and select the 'Submissions Needing Revision' folder to locate your manuscript file.

- A letter that responds to each point raised by the academic editor and reviewer(s). You should upload this letter as a separate file labeled 'Response to Reviewers'.
- A marked-up copy of your manuscript that highlights changes made to the original version. You should upload this as a separate file labeled 'Revised Manuscript with Track Changes'.
- An unmarked version of your revised paper without tracked changes. You should upload this as a separate file labeled 'Manuscript'.

If you would like to make changes to your financial disclosure, please include your updated statement in your cover letter. Guidelines for resubmitting your figure files are available below the reviewer comments at the end of this letter.

If applicable, we recommend that you deposit your laboratory protocols in protocols.io to enhance the reproducibility of your results. Protocols.io assigns your protocol its own identifier (DOI) so that it can be cited independently in the future. For instructions see: https://journals.plos.org/plosone/s/submission-guidelines#loc-laboratory-protocols. Additionally, PLOS ONE offers an option for publishing peer-reviewed Lab Protocol articles, which describe protocols hosted on protocols.io. Read more information on sharing protocols at https://plos.org/protocols?utm_medium=editorial-email&utm_source=authorletters&utm_campaign=protocols.

As the corresponding author, your ORCID iD is verified in the submission system and will appear in the published article. PLOS supports the use of ORCID, and we encourage all coauthors to register for an ORCID iD and use it as well. Please encourage your coauthors to verify their ORCID iD within the submission system before final acceptance, as unverified ORCID iDs will not appear in the published article. *Only*  the individual author can complete the verification step; PLOS staff *cannot*  verify ORCID iDs on behalf of authors.

We look forward to receiving your revised manuscript.

Kind regards,

Giovanni Nassa, PhD

Academic Editor

PLOS One

Journal Requirements:

When submitting your revision, we need you to address these additional requirements.

1. Please ensure that your manuscript meets PLOS ONE's style requirements, including those for file naming. The PLOS ONE style templates can be found at https://journals.plos.org/plosone/s/file?id=wjVg/PLOSOne_formatting_sample_main_body.pdf and https://journals.plos.org/plosone/s/file?id=ba62/PLOSOne_formatting_sample_title_authors_affiliations.pdf

2. We noticed you have some minor occurrence of overlapping text with the following previous publication(s), which needs to be addressed:

Integrated proteomics identifies PARP inhibitor-induced prosurvival signaling changes as potential vulnerabilities in ovarian cancer - 10.1016/j.jbc.2022.102550

In your revision ensure you cite all your sources (including your own works), and quote or rephrase any duplicated text outside the methods section. Further consideration is dependent on these concerns being addressed.

3. Thank you for stating in your Funding Statement:

[This project was supported by a Hearing the Ovarian Cancer Whisper Jacquie Liggett Fellowship (to O.D.), the Sarasota foundation fund (to A. N. M.) and the NIH/NCI R21 CA252373 (to A. N. M. and U. R.). Moffitt’s Proteomics & Metabolomics, Molecular Genomics and Chemical Biology Cores are funded in part by the National Cancer Institute through the Cancer Center Support Grant (P30-CA076292), which confers Moffitt’s status as a comprehensive cancer center.].

Please provide an amended statement that declares *all* the funding or sources of support (whether external or internal to your organization) received during this study, as detailed online in our guide for authors at http://journals.plos.org/plosone/s/submit-now.  Please also include the statement “There was no additional external funding received for this study.” in your updated Funding Statement.

Please include your amended Funding Statement within your cover letter. We will change the online submission form on your behalf.

4. Please remove any funding-related text from the manuscript. Please note that funding information should not appear in any section or other areas of your manuscript. We will publish the funding statement that is present in the Financial Disclosure section of the online submission form.

5. PLOS ONE now requires that authors provide the original uncropped and unadjusted images underlying all blot or gel results reported in a submission’s figures or Supporting Information files. This policy and the journal’s other requirements for blot/gel reporting and figure preparation are described in detail at https://journals.plos.org/plosone/s/figures#loc-blot-and-gel-reporting-requirements and https://journals.plos.org/plosone/s/figures#loc-preparing-figures-from-image-files. When you submit your revised manuscript, please ensure that your figures adhere fully to these guidelines and provide the original underlying images for all blot or gel data reported in your submission. See the following link for instructions on providing the original image data: https://journals.plos.org/plosone/s/figures#loc-original-images-for-blots-and-gels.

In your cover letter, please note whether your blot/gel image data are in Supporting Information or posted at a public data repository, provide the repository URL if relevant, and provide specific details as to which raw blot/gel images, if any, are not available. Email us at plosone@plos.org if you have any questions.

5. Please upload a new copy of Figure 1-6 as the detail is not clear. Please follow the link for more information:  https://journals.plos.org/plosone/s/figures

6. Please include captions for all your Supporting Information files at the end of your manuscript, and update any in-text citations to match accordingly. Please see our Supporting Information guidelines for more information: http://journals.plos.org/plosone/s/supporting-information.

If the reviewer comments include a recommendation to cite specific previously published works, please review and evaluate these publications to determine whether they are relevant and should be cited. There is no requirement to cite these works unless the editor has indicated otherwise.

Please review your reference list to ensure that it is complete and correct. If you have cited papers that have been retracted, please include the rationale for doing so in the manuscript text, or remove these references and replace them with relevant current references. Any changes to the reference list should be mentioned in the rebuttal letter that accompanies your revised manuscript. If you need to cite a retracted article, indicate the article’s retracted status in the References list and also include a citation and full reference for the retraction notice.

[Note: HTML markup is below. Please do not edit.]

Reviewers' comments:

Reviewer's Responses to Questions

**Comments to the Author**

1. Is the manuscript technically sound, and do the data support the conclusions?

Reviewer #1: Yes

Reviewer #2: Yes

**********

2. Has the statistical analysis been performed appropriately and rigorously?

Reviewer #1: Yes

Reviewer #2: I Don't Know

**********

3. Have the authors made all data underlying the findings in their manuscript fully available?

The PLOS Data policy

Reviewer #1: Yes

Reviewer #2: Yes

**********

4. Is the manuscript presented in an intelligible fashion and written in standard English?

Reviewer #1: Yes

Reviewer #2: Yes

**********

Reviewer #1: The manuscript is interesting and provides useful insights into mechanisms of PARPi sensitivity in BRCA-proficient ovarian cancer. The proteomics analysis is a clear strength of the study. The work is generally well performed, but several points could be clarified to further strengthen the manuscript.

Main Comments

1. All experiments were performed in cell lines. This limitation should be discussed more clearly. Validation in xenograft, organoid, or patient-derived models would further support the translational relevance of the findings, if feasible.

2. The connection between MSH6 and CHAF1A is interesting, but currently appears mostly correlative. The manuscript would benefit from additional discussion of:

MSH6 regulates CHAF1A directly,

CHAF1A is required for MSH6-related PARPi

rescue experiments were considered or attempted.

3. The decrease in CHAF1A ADP-ribosylation is interesting, but it is not fully clear whether this reflects a specific mechanism or broader changes in PARP activity. Some mechanistic conclusions may need to be stated more cautiously.

4. The classification of “sensitive” and “resistant” based mainly on rucaparib Cmax may oversimplify biological differences between models. Additional discussion of HRD status, replication stress, genomic instability, or published PARPi response data would strengthen the interpretation.

5. Since MSH6 is an important mismatch repair protein, the Discussion should more fully address mismatch repair deficiency, microsatellite instability, and their possible relationship with PARPi response.

6. More detail on statistical analysis would improve clarity, including:

multiple testing correction,

exact p-values or effect sizes,

and justification of proteomics filtering thresholds.

Minor Comments

1. Some figure legends are too long and could be simplified.

2. In several places, associative wording may be more appropriate than causal wording.

3. The Discussion would benefit from additional comparison with previous studies on PARP1, mismatch repair, and replication fork stability.

4. Minor grammar and formatting issues should be corrected.

5. Abbreviations should be used consistently throughout the manuscript.

Questions for the Authors

1. Were rescue experiments with CHAF1A restoration considered or performed?

2. Are there public datasets linking MSH6 expression with PARPi response in BRCA-proficient ovarian cancer?

3. Have the authors considered validation in organoid or in vivo models?

4. Did the authors examine HR-related markers such as RAD51 foci after MSH6 depletion?

5. How did the authors distinguish specific PARylation effects from global PARP inhibition effects?

Overall Assessment

Overall, this is a strong and potentially impactful study. The manuscript would be further improved by additional clarification, expanded discussion of limitations, and more cautious interpretation of mechanistic conclusions.

Reviewer #2: The study investigated the mechanisms of PARP inhibitor (PARPi) sensitivity in BRCA-proficient ovarian cancer. The methods are sophisticated and appropriate. The multi-platform proteomic workflow combined with molecular biological validation is a major strength and appears technically rigorous. Data supports the conclusion. The identification of a PARP1/MSH6/CHAF1A-associated network linked to PARPi sensitivity is potentially novel and clinically relevant. Given the need for biomarkers for PARPi responses beyond BRCA deficiency, this research is highly interesting.

There are a few comments that need to be discussed.

In the survival analysis (Fig 4), stratified analysis in patients receiving platinum and taxol based chemotherapy were analyzed. How about patients receiving PARPi? Is MSH6 related to clinical outcomes of patients receiving PAPRi?

The proposed role of CHAF1A PARylation is largely associative. Direct evidence is lacking demonstrating functional dependence on CHAF1A PARylation sites.

The translational relevance is promising but a bit overstated. Claims regarding biomarker potential for MSH6 and PARP1 should be tempered because patient survival analyses are modest and largely indirect.

Overall, this is a strong and potentially impactful study, but additional mechanistic validation and more cautious interpretation are needed before publication in a high-impact setting.

A minor comment:

Line 296-297: “…only rucaparib was FDA-approved as maintenance therapy for recurrent OC regardless of BRCA mutation status” is not accurate. For example, niraparib also is FAD-approved for maintenance therapy regardless of BRCA/HRD status.

**********

what does this mean?). If published, this will include your full peer review and any attached files.

If you choose “no”, your identity will remain anonymous but your review may still be made public.

**Do you want your identity to be public for this peer review?**  For information about this choice, including consent withdrawal, please see our Privacy Policy

Reviewer #1: **Yes:** Raikhan Bolatbekova

Reviewer #2: **Yes:** Qi Chen

**********

[NOTE: If reviewer comments were submitted as an attachment file, they will be attached to this email and accessible via the submission site. Please log into your account, locate the manuscript record, and check for the action link "View Attachments". If this link does not appear, there are no attachment files.]

To ensure your figures meet our technical requirements, please review our figure guidelines: https://journals.plos.org/plosone/s/figures

You may also use PLOS’s free figure tool, NAAS, to help you prepare publication quality figures: https://journals.plos.org/plosone/s/figures#loc-tools-for-figure-preparation.

NAAS will assess whether your figures meet our technical requirements by comparing each figure against our figure specifications.

# Author comment: Author response to Decision Letter 1

**DOI:** 10.1371/journal.pone.0357365.r002

6 Jul 2026

Response to Academic Editor

General Editorial Assessment

Comment:

The manuscript appears scientifically solid, technically rigorous, and potentially impactful, with strengths in the proteomics workflow and translational relevance. However, several concerns were raised regarding mechanistic validation, interpretation, and clinical relevance, which require additional discussion and textual revision.

Response:

We sincerely thank the Academic Editor for the positive evaluation of our work and for the constructive guidance. In response to these concerns, we substantially revised the manuscript to strengthen the discussion of mechanistic validation, clarify interpretation of the findings, and better contextualize the clinical relevance of the study. Specifically, we:

Expanded the Discussion section regarding mechanistic implications and limitations;

Clarified the interpretation of the proteomics findings;

Added discussion addressing the suggested future validation experiments, including CHAF1A rescue studies, PARylation site validation, and in vivo/organoid validation approaches.

These revisions are reflected throughout the revised manuscript.

Journal Requirements

Comment 1

Please ensure that your manuscript meets PLOS ONE style requirements, including file naming and formatting.

Response:

We carefully revised the manuscript to comply with all PLOS ONE formatting and file naming requirements.

Comment 2

We noticed you have some minor occurrence of overlapping text with the following previous publication(s), which needs to be addressed:

Integrated proteomics identifies PARP inhibitor-induced prosurvival signaling changes as potential vulnerabilities in ovarian cancer - 10.1016/j.jbc.2022.102550

In your revision ensure you cite all your sources (including your own works), and quote or rephrase any duplicated text outside the methods section. Further consideration is dependent on these concerns being addressed.

Response:

We thank the editor for bringing this to our attention. We carefully reviewed our manuscript in comparison with the cited publication (DOI: 10.1016/j.jbc.2022.102550), which was already cited in the Introduction:

“In our previous work, we have observed that the composition of PARP1-associated protein complexes and patterns of poly-ADP-ribosylation (PARylation) may play critical roles in differentially modulating PARPi response between BRCA-deficient and BRCA-proficient OC cells [20].”

In addition, we reviewed the manuscript for potential text overlap outside the Methods section and revised the wording where appropriate. We have also verified that all relevant prior work, including our own publications, is properly cited throughout the manuscript.

Comment 3

Please amend the Funding Statement and include: “There was no additional external funding received for this study.”

Response:

We updated the Funding Statement to include all funding sources and added the requested statement: “There was no additional external funding received for this study.”

Comment 4

Please remove funding-related text from the manuscript.

Response:

All funding-related text has been removed from the manuscript body in accordance with PLOS ONE guidelines.

Comment 5

Please provide original uncropped and unadjusted blot/gel images.

Response:

We have provided the original uncropped and unadjusted blot/gel images as Supporting Information files, in accordance with PLOS ONE figure reporting requirements.

Comment 6

Please upload higher-quality versions of Figures 1–6.

Response:

We uploaded revised high-resolution versions of Figures 1–6 to improve image clarity and readability.

Comment 7

Please include captions for all Supporting Information files and update in-text citations accordingly.

Response:

Captions for all Supporting Information files have been added at the end of the manuscript, and all in-text citations have been updated accordingly.

Response to Reviewer #1

Main Comments Comment 1 All experiments were performed in cell lines. This limitation should be discussed more clearly. Validation in xenograft, organoid, or patient-derived models would further support the translational relevance of the findings, if feasible. Response: We appreciate the reviewer’s insightful comment and agree that validation in organoid, xenograft, or patient-derived models would further strengthen the translational relevance of our findings. We have therefore revised the Discussion section to explicitly acknowledge this limitation and emphasize the importance of future validation in physiologically relevant systems. Accordingly, we have added this aspect to the discussion:

“A limitation of the present study is that it is primarily based on established ovarian cancer cell line models. Therefore, further validation experiments will also have to be performed in patient-derived organoids, xenografts, and clinical tumor specimens to establish its translational relevance and potential clinical applicability.” (Page 32)

Comment 2 The connection between MSH6 and CHAF1A is interesting, but currently appears mostly correlative. The manuscript would benefit from additional discussion of:

MSH6 regulates CHAF1A directly,

CHAF1A is required for MSH6-related PARPi

rescue experiments were considered or attempted.

Response: We appreciate this important observation. In the revised manuscript, we toned down mechanistic interpretations regarding the relationship between MSH6 and CHAF1A and clarified that our current findings support a functional association rather than definitive direct regulation. We further expanded the Discussion to acknowledge that the precise mechanistic relationship between MSH6 and CHAF1A remains to be established, and that rescue experiments and targeted mechanistic validation would be valuable future directions to clarify the functional hierarchy within this pathway and to define its contribution to PARPi sensitivity in BRCA-proficient ovarian cancer. Accordingly, we have added this to the discussion:

“Although our data identify the pair-wise interactions between PARP1, MSH6 and CHAF1A and illustrate the functional effects of MSH6 on CHAF1A protein levels and PARPi sensitivity, the precise mechanistic relationship between these proteins remains to be defined. It is currently unclear whether MSH6 directly regulates CHAF1A activity or whether CHAF1A also contributes to modulating PARPi response. Further functional studies, including rescue experiments and targeted perturbation approaches, will be required to clarify these signaling mechanisms in detail.” (Page 32)

Comment 3 The decrease in CHAF1A ADP-ribosylation is interesting, but it is not fully clear whether this reflects a specific mechanism or broader changes in PARP activity. Response:

We agree that the observed decrease in CHAF1A ADP-ribosylation should be interpreted cautiously. Our study was designed to compare ADP-ribosylation profiles between PARPi-sensitive and -resistant cells. Following rucaparib treatment, we observed a significant reduction in CHAF1A ADP-ribosylation, and CHAF1A emerged as one of the proteins exhibiting differential ADP-ribosylation between PARPi-sensitive and -resistant cells. Considering the pronounced effects of CHAF1A silencing on cell viability, these findings suggest that CHAF1A may represent a downstream target associated with PARP1 signaling and PARPi response.

However, future studies will be important to determine the extent to which CHAF1A ADP-ribosylation contributes to PARPi sensitivity and therapeutic response. Additional mechanistic studies, including PARP1 knockout or depletion models, biochemical validation of CHAF1A ADP-ribosylation, and site-specific ADP-ribosylation analyses, will be required to further investigate the regulation of CHAF1A ADP-ribosylation. Accordingly, we have revised the Discussion to tone down the mechanistic interpretation and clarify these limitations:

“These findings indicate that CHAF1A ADP-ribosylation is associated with PARPi response and may contribute to the differential assembly of the PARP1/MSH6/CHAF1A complex.

However, whether CHAF1A is directly ADP-ribosylated by PARP1 or other known PARP family targets, such as PARP2 or tankyrase [37, 68], ADP-ribosylation occurs at specific functional sites or contributes directly to CHAF1A’s regulatory activity requires further studies using PARP1 knockdown, site-directed mutagenesis and mapping of CHAF1A PARylation sites.” (Page 30)

“However, the functional consequences of CHAF1A ADP-ribosylation, its impact on protein interactions and PARPi response were not examined in this study and will require further investigation.” (Page 31)

Comment 4 The classification of “sensitive” and “resistant” based mainly on rucaparib Cmax may oversimplify biological differences between models. Response:

We appreciate the reviewer’s concern. However, the classification of PARPi-sensitive and -resistant models was not based solely on rucaparib Cmax. PARPi sensitivity was evaluated according to the IC50 values of all three FDA-approved PARPis across the BRCA-proficient ovarian cancer cell lines.

As described in Results section 1, we focused on rucaparib because it is the PARPi FDA-approved for recurrent ovarian cancer regardless of BRCA status. At the clinically relevant Cmax (1.94 μM), only about one-third of the tested cell lines showed >50% reduction in viability, while most exhibited limited response.

Based on these response patterns, we selected A2780, OVCAR8, and Caov3 as representative PARPi-sensitive lines and HEYA8, SKOV3, and OVCA432 as PARPi-resistant lines (Fig. 1B). OVCAR3 was excluded despite its sensitivity because of previously reported deep BRCA2 deletions, which could confound interpretation of BRCA-proficient PARPi responses.

We have now rewritten this sentence to reflect the sensitivity/resistance to the other PARPis more clearly, now reads (changes in bold):

“Based on their IC50 values, relative to rucaparib’s maximal plasma concentration (1.94 mM), and consistency across the other PARPis olaparib and niraparib, cell lines were classified as sensitive or resistant (Fig 1B).” (Page 14)

Comment 5 Since MSH6 is an important mismatch repair protein, the Discussion should more fully address mismatch repair deficiency, microsatellite instability, and their possible relationship with PARPi response. Response:

We thank the reviewer for this important suggestion. We agree that MSH6 is a core component of the DNA mismatch repair (MMR) pathway and is frequently discussed in

the context of mismatch repair deficiency and microsatellite instability (MSI), which have been associated with genome instability and therapeutic responses in cancer.

However, MSI-high ovarian cancers are relatively uncommon, and the majority of ovarian cancers, including the models used in our study, are microsatellite stable (MSS). Therefore, the biological context of our study differs from that of MSI-associated malignancies in which MSH6 dysfunction has been more extensively investigated.

Previous studies investigating MSH6 in the context of PARP inhibitor response have largely focused on MMR deficiency, MSI, or genetic alterations of MMR pathway components. In contrast, the ovarian cancer models examined in the present study are predominantly microsatellite stable (MSS) and BRCA-proficient. Therefore, our findings relate to differential MSH6 protein expression and protein complex formation rather than classical MMR deficiency. To our knowledge, the association between MSH6 protein expression and PARP inhibitor sensitivity in BRCA-proficient ovarian cancer has not been systematically investigated.

We have revised the Discussion to explicitly acknowledge the canonical role of MSH6 in MMR and its established relationship with MSI. However, our experimental findings suggest that MSH6 may also have functions beyond its traditional role in mismatch repair. Notably, depletion of MSH6 did not significantly affect cell viability, indicating that the observed effects are unlikely to be explained solely by loss of canonical MMR activity. Instead, our data supports a model in which MSH6 contributes to PARP inhibitor response through altered protein interactions, including changes in PARP1-associated complexes and downstream signaling pathways. Nevertheless, the precise mechanisms linking MSH6 expression to PARP inhibitor sensitivity remain to be fully elucidated and warrant further investigation. These revisions have been incorporated into the Discussion:

“MSH6 has been extensively studied in the context of MMR deficiency and microsatellite instability (MSI) [57-60]. However, MSI-high tumors are relatively uncommon in ovarian cancer, occurring in approximately 1–3% of serous cases [61-65]. Although MSI status was not directly assessed in the present study, previous reports have classified SKOV3 as MSI-H, whereas the remaining cell lines used in this study are generally considered microsatellite stable (MSS) [66, 67]. Therefore, the differential MSH6 expression and PARP1/MSH6 complex formation observed in our models are unlikely to be explained solely by classical MMR deficiency. Instead, these findings support the possibility that MSH6 may have additional functions beyond canonical mismatch repair, potentially through PARP1-associated signaling pathways that influence PARPi response. The relationship between MSH6-associated signaling, MSI phenotypes, and PARPi sensitivity warrants further investigation.” (Page 29-30)

Comment 6 More detail on statistical analysis would improve clarity.

Response:

Additional details regarding statistical analyses have now been included in the Methods section:

“Data was obtained from at least three independent experiments and shown as the mean ± SD. Data was analyzed using Microsoft Excel and GraphPad Prism 10.0. Statistical analyses were performed using Student’s t test (paired or unpaired, as appropriate) and synergy was determined by the Bliss independence model[35]. P value of <0.05 was considered statistically significant. All statistical details are included in the figure legends. All MaxQuant data were first filtered for peptides with PEP score < 0.05. Furthermore, reverse and contaminant peptides and peptides with no intensity were excluded. Data were then normalized using IRON[41].” (Page 13) Minor Comments

1. Some figure legends are too long and could be simplified.

2. In several places, associative wording may be more appropriate than causal wording.

3. The Discussion would benefit from additional comparison with previous studies on PARP1, mismatch repair, and replication fork stability.

4. Minor grammar and formatting issues should be corrected.

5. Abbreviations should be used consistently throughout the manuscript.

Response: We thank the reviewer for these suggestions. Figure legends were revised for clarity and conciseness, causal wording was modified where appropriate, additional literature comparisons were incorporated into the Discussion, and the manuscript was carefully edited for grammar, formatting consistency, and standardized abbreviation usage throughout. Questions for the Authors

Question 1 Were rescue experiments with CHAF1A restoration considered or performed? Response: We agree that such experiments would provide important mechanistic validation and would have liked to engage in these experiments:

“A limitation of the present study is that it is primarily based on established ovarian cancer cell line models. Therefore, further validation experiments will also have to be performed in patient-derived organoids, xenografts, and clinical tumor specimens to establish its translational relevance and potential clinical applicability.” (Page 32) Question 2

Are there public datasets linking MSH6 expression with PARPi response in BRCA-proficient ovarian cancer? Response: To the best of our knowledge, as PARPis have been approved for ovarian cancer only for a few years so far and not as first-line therapy, there are currently no large, dedicated public clinical datasets that directly stratify PARPi response by MSH6 expression specifically in BRCA-proficient ovarian cancer cohorts.

Question 3 Have the authors considered validation in organoid or in vivo models? Response: Yes. We agree that validation in organoid and in vivo systems would substantially st

Attachment Submitted filename: Response to Reviewers.pdf

# Peer review: Decision Letter 1

**DOI:** 10.1371/journal.pone.0357365.r003

**Authors:** Giovanni Nassa

29 Jul 2026

Dear Dr. Deng,

Thank you for submitting your manuscript to PLOS ONE. After careful consideration, we feel that it has merit but does not fully meet PLOS ONE’s publication criteria as it currently stands. Therefore, we invite you to submit a revised version of the manuscript that addresses the points raised during the review process.

Please submit your revised manuscript by Sep 12 2026 11:59PM. If you will need more time than this to complete your revisions, please reply to this message or contact the journal office at plosone@plos.org. When you're ready to submit your revision, log on to https://www.editorialmanager.com/pone/ and select the 'Submissions Needing Revision' folder to locate your manuscript file.

- A letter that responds to each point raised by the academic editor and reviewer(s). You should upload this letter as a separate file labeled 'Response to Reviewers'.
- A marked-up copy of your manuscript that highlights changes made to the original version. You should upload this as a separate file labeled 'Revised Manuscript with Track Changes'.
- An unmarked version of your revised paper without tracked changes. You should upload this as a separate file labeled 'Manuscript'.

If you would like to make changes to your financial disclosure, please include your updated statement in your cover letter. Guidelines for resubmitting your figure files are available below the reviewer comments at the end of this letter.

If applicable, we recommend that you deposit your laboratory protocols in protocols.io to enhance the reproducibility of your results. Protocols.io assigns your protocol its own identifier (DOI) so that it can be cited independently in the future. For instructions see: https://journals.plos.org/plosone/s/submission-guidelines#loc-laboratory-protocols. Additionally, PLOS ONE offers an option for publishing peer-reviewed Lab Protocol articles, which describe protocols hosted on protocols.io. Read more information on sharing protocols at https://plos.org/protocols?utm_medium=editorial-email&utm_source=authorletters&utm_campaign=protocols.

As the corresponding author, your ORCID iD is verified in the submission system and will appear in the published article. PLOS supports the use of ORCID, and we encourage all coauthors to register for an ORCID iD and use it as well. Please encourage your coauthors to verify their ORCID iD within the submission system before final acceptance, as unverified ORCID iDs will not appear in the published article. *Only*  the individual author can complete the verification step; PLOS staff *cannot*  verify ORCID iDs on behalf of authors.

We look forward to receiving your revised manuscript.

Kind regards,

Giovanni Nassa, PhD

Academic Editor

PLOS One

Journal Requirements:

If the reviewer comments include a recommendation to cite specific previously published works, please review and evaluate these publications to determine whether they are relevant and should be cited. There is no requirement to cite these works unless the editor has indicated otherwise.

Please review your reference list to ensure that it is complete and correct. If you have cited papers that have been retracted, please include the rationale for doing so in the manuscript text, or remove these references and replace them with relevant current references. Any changes to the reference list should be mentioned in the rebuttal letter that accompanies your revised manuscript. If you need to cite a retracted article, indicate the article’s retracted status in the References list and also include a citation and full reference for the retraction notice.

**Additional Editor Comments:**

Overall, now the manuscript is a well-conducted and impactful study. Only a few minor points require clarification to further strengthen the mechanistic interpretation and improve the manuscript's clarity as requested by R1.

[Note: HTML markup is below. Please do not edit.]

Reviewers' comments:

Reviewer's Responses to Questions

**Comments to the Author**

Reviewer #1: All comments have been addressed

Reviewer #2: All comments have been addressed

**********

2. Is the manuscript technically sound, and do the data support the conclusions?

Reviewer #1: Yes

Reviewer #2: Yes

**********

3. Has the statistical analysis been performed appropriately and rigorously?

Reviewer #1: Yes

Reviewer #2: I Don't Know

**********

4. Have the authors made all data underlying the findings in their manuscript fully available?

The PLOS Data policy

Reviewer #1: Yes

Reviewer #2: Yes

**********

5. Is the manuscript presented in an intelligible fashion and written in standard English?

Reviewer #1: Yes

Reviewer #2: Yes

**********

Reviewer #1: The study addresses a critical clinical challenge in gynecologic oncology: expanding PARP inhibitor (PARPi) utility to BRCA-proficient tumors. The multi-omics framework—coupling chemical proteomics (rucaparib pull-downs) with ADP-ribosylation proteomics—is innovative and technically robust.

Primary Conceptual Gap: The mechanistic transition between MSH6 loss and mTOR activation needs clearer bridging. The text posits that CHAF1A acts downstream of MSH6 to regulate mTOR, but the exact mechanism remains speculative. Clarify this boundary in the Discussion.

Title & Abstract

Clear, well-structured, and accurately reflects the scope of the manuscript.

Introduction

Line 74–75: Clarify the criteria for "BRCA-proficient" upfront (e.g., absence of somatic/germline pathogenic mutations and functional HR proficiency where validated).

Materials and Methods

Line 88: Standardize cell line naming: ensure consistency between HEAYA8 (Line 85), HEAY8 (Line 353), and HEYA8 (used elsewhere). Standardize to HEYA8 across all sections

Results

Selecting A2780, OVCAR8, and Caov3 as sensitive lines versus HEYA8, SKOV3, and OVCA432 as resistant lines is well-justified.

A2780 is widely acknowledged as an endometrioid/undifferentiated line rather than classic high-grade serous ovarian carcinoma (HGSOC). While acceptable for general BRCA-proficient OC modeling, add a brief parenthetical note or statement in the Discussion clarifying cell line histotypes.

Highlight the novelty of MSH6 enrichment over MSH2, which suggests an unconventional MMR-independent assembly.

Discussion

Consider adding a concise summary paragraph at the start of the Discussion that explicitly contrasts sensitive versus resistant states:

Sensitive cells: High MSH6 → PARP1–MSH6–CHAF1A complex intact → PARP1 inhibition reduces CHAF1A PARylation → suppression of mTOR signaling → cell death/synthetic lethality.

Resistant cells: Low MSH6 / complex dissociation → elevated CHAF1A → compensatory mTORC1 pathway activation → survival (targetable with mTOR/S6K inhibitors).

Study Limitations: Expand slightly on the limitations, specifically noting the need for future in vivo PDX/allograft validation and broader clinical cohort verification for MSH6/PARP1 co-expression as a predictive biomarker.

Reviewer #2: The authors have addressed all previously critiques. There is no additional comments. I recommend acceptance for publication.

**********

what does this mean?). If published, this will include your full peer review and any attached files.

If you choose “no”, your identity will remain anonymous but your review may still be made public.

**Do you want your identity to be public for this peer review?**  For information about this choice, including consent withdrawal, please see our Privacy Policy

Reviewer #1: No

Reviewer #2: **Yes:** Qi Chen

**********

[NOTE: If reviewer comments were submitted as an attachment file, they will be attached to this email and accessible via the submission site. Please log into your account, locate the manuscript record, and check for the action link "View Attachments". If this link does not appear, there are no attachment files.]

To ensure your figures meet our technical requirements, please review our figure guidelines: https://journals.plos.org/plosone/s/figures

You may also use PLOS’s free figure tool, NAAS, to help you prepare publication quality figures: https://journals.plos.org/plosone/s/figures#loc-tools-for-figure-preparation.

NAAS will assess whether your figures meet our technical requirements by comparing each figure against our figure specifications.

# Author comment: Author response to Decision Letter 2

**DOI:** 10.1371/journal.pone.0357365.r004

14 Aug 2026

Response to Reviewer #1

We sincerely thank the reviewer for the thoughtful and constructive comments. We appreciate the positive assessment of our study and have revised the manuscript accordingly. Our point-by-point responses are provided below.

Comment 1. The mechanistic transition between MSH6 loss and mTOR activation needs clearer bridging. The text posits that CHAF1A acts downstream of MSH6 to regulate mTOR, but the exact mechanism remains speculative. Clarify this boundary in the Discussion.

Response:

We thank the reviewer for this important comment. We agree that the mechanistic relationship between MSH6 loss, CHAF1A regulation, and mTOR activation remains incompletely defined. We have revised the Discussion to clarify this mechanistic boundary. The revised text now reads:

“These findings link MSH6, probably through modulation of CHAF1A–mTOR signaling, to PARPi resistance in BRCA-proficient OC. However, whether and how CHAF1A is directly regulated by MSH6 or serves as a critical mediator connecting MSH6 loss to mTOR activation remains to be determined.” (Page 31)

Comment 2. Line 74–75: Clarify the criteria for "BRCA-proficient" upfront (e.g., absence of somatic/germline pathogenic mutations and functional HR proficiency where validated).

Response:

We thank the reviewer for this suggestion. We have clarified the criteria for "BRCA-proficient" in the Introduction. The revised text now reads:

“In this study, we investigated the proteomic landscape associated with PARPi sensitivity in a diverse panel of BRCA-proficient OC cell lines, defined here by the absence of BRCA1/2 mutations or genomic deletions. Most cell lines used in this study have previously been reported to exhibit HR proficiency [21-24].” (Page 4)

Comment 3. Line 88: Standardize cell line naming: ensure consistency between HEAYA8 (Line 85), HEAY8 (Line 353), and HEYA8 (used elsewhere). Standardize to HEYA8 across all sections

Response:

We thank the reviewer for identifying this inconsistency. The manuscript has been carefully reviewed, and all instances have been corrected to the standardized cell line name, HEYA8, throughout the manuscript.

Comment 4.

Results

Selecting A2780, OVCAR8, and Caov3 as sensitive lines versus HEYA8, SKOV3, and OVCA432 as resistant lines is well-justified.

A2780 is widely acknowledged as an endometrioid/undifferentiated line rather than classic high-grade serous ovarian carcinoma (HGSOC). While acceptable for general BRCA-proficient OC modeling, add a brief parenthetical note or statement in the Discussion clarifying cell line histotypes.

Response:

We appreciate this important comment. A brief statement has been added to the Discussion acknowledging that A2780 is an endometrioid/undifferentiated ovarian cancer cell line. The revised text now reads:

“To further investigate the mechanisms underlying PARPi sensitivity and resistance, we employed chemical proteomics and ADP-ribosylation proteomics to examine PARP1 protein complexes and PARylation profiles in PARPi-sensitive and -resistant BRCA-proficient OC cells, including the endometrioid OC cell line A2780.” (Page 28)

Comment 5. Highlight the novelty of MSH6 enrichment over MSH2, which suggests an unconventional MMR-independent assembly.

Response:

We thank the reviewer for this insightful comment. We agree that the selective enrichment of MSH6 over MSH2 represents an important observation of our study. We have revised the Discussion to further emphasize the potential significance of this finding. The revised text now reads:

“Although PARP1 and MSH6 have been previously reported to interact during mismatch repair (MMR)[55, 56], particularly during the recognition and signaling of DNA mismatches, our chemical proteomics data demonstrated selective enrichment of PARP1/MSH6 complexes in PARPi-sensitive cells with no apparent enrichment of MSH2. This finding suggests an unconventional, potentially MMR-independent role for MSH6 in modulating PARPi sensitivity.” (Page 29)

Comment 6.

Discussion

Consider adding a concise summary paragraph at the start of the Discussion that explicitly contrasts sensitive versus resistant states:

Sensitive cells: High MSH6 → PARP1–MSH6–CHAF1A complex intact → PARP1 inhibition reduces CHAF1A PARylation → suppression of mTOR signaling → cell death/synthetic lethality.

Resistant cells: Low MSH6 / complex dissociation → elevated CHAF1A → compensatory mTORC1 pathway activation → survival (targetable with mTOR/S6K inhibitors).

Response:

We appreciate this suggestion. We have added a concise summary paragraph at the beginning of the Discussion. The new paragraph summarizes the differences in MSH6-associated PARP1 complex formation, CHAF1A ADP-ribosylation and expression, mTOR signaling, and cellular response to rucaparib, and highlights the potential therapeutic relevance of mTOR pathway inhibition in PARPi-resistant cells.

“Overall, our findings support a model in which PARPi-sensitive and PARPi-resistant BRCA-proficient OC cells exhibit distinct PARP1-associated protein complexes and downstream signaling. PARPi-sensitive compared to PARPi-resistant cells preferentially featured a PARP1-MSH6-CHAF1A complex and higher CHAF1A ADP-ribosylation, reduced by PARPi treatment, which prevented cells to launch PARPi-adaptive mTOR survival signaling. In contrast, PARPi-resistant cells showed compensatory activation of mTOR signaling following PARPi treatment and enhancement of rucaparib effects by pharmacological inhibition of the mTOR pathway. These findings suggest that compensatory mTOR signaling contributes to PARPi resistance and that mTOR pathway inhibition may be a potential combination strategy for PARPi-resistant cells.” (Page 28)

Comment 7. Study Limitations: Expand slightly on the limitations, specifically noting the need for future in vivo PDX/allograft validation and broader clinical cohort verification for MSH6/PARP1 co-expression as a predictive biomarker.

Response:

We thank the reviewer for this valuable suggestion. We have expanded the Limitations section to further acknowledge the need for in vivo validation and evaluation of MSH6/PARP1 co-expression as a predictive biomarker for PARPi response.

The revised text now reads:

“A limitation of the present study is that it is primarily based on established ovarian cancer cell line models. Therefore, further validation experiments will also have to be performed in patient-derived xenografts (PDX) and organoids (PDO), as well as clinical tumor specimens to establish its translational relevance and potential clinical applicability. In particular, evaluation of MSH6/PARP1 co-expression in larger clinical cohorts will be required to determine its potential utility as a predictive biomarker for PARPi response.” (Page 32)

Attachment Submitted filename: Response_to_Reviewers_auresp_2.pdf

# Editor report: Decision Letter 2

**DOI:** 10.1371/journal.pone.0357365.r005

**Authors:** Giovanni Nassa

17 Aug 2026

Integrative proteomics reveals MSH6 to modulate PARP inhibitor sensitivity in BRCA1/2-proficient ovarian cancer

PONE-D-26-10571R2

Dear Dr. Deng,

We’re pleased to inform you that your manuscript has been judged scientifically suitable for publication and will be formally accepted for publication once it meets all outstanding technical requirements.

Within one week, you’ll receive an e-mail detailing the required amendments. When these have been addressed, you’ll receive a formal acceptance letter and your manuscript will be scheduled for publication.

An invoice will be generated when your article is formally accepted. Please note, if your institution has a publishing partnership with PLOS and your article meets the relevant criteria, all or part of your publication costs will be covered. Please make sure your user information is up-to-date by logging into Editorial Manager at Editorial Manager® and clicking the ‘Update My Information' link at the top of the page. For questions related to billing, please contact billing support.

If your institution or institutions have a press office, please notify them about your upcoming paper to help maximize its impact. If they’ll be preparing press materials, please inform our press team as soon as possible -- no later than 48 hours after receiving the formal acceptance. Your manuscript will remain under strict press embargo until 2 pm Eastern Time on the date of publication. For more information, please contact onepress@plos.org.

Kind regards,

Giovanni Nassa, PhD

Academic Editor

PLOS One

Additional Editor Comments (optional):

Reviewers' comments:

# Editor report: Acceptance letter

**DOI:** 10.1371/journal.pone.0357365.r006

**Authors:** Giovanni Nassa

PONE-D-26-10571R2

PLOS One

Dear Dr. Deng,

I'm pleased to inform you that your manuscript has been deemed suitable for publication in PLOS One. Congratulations! Your manuscript is now being handed over to our production team.

At this stage, our production department will prepare your paper for publication. This includes ensuring the following:

* All references, tables, and figures are properly cited

* All relevant supporting information is included in the manuscript submission,

* There are no issues that prevent the paper from being properly typeset

You will receive further instructions from the production team, including instructions on how to review your proof when it is ready. Please keep in mind that we are working through a large volume of accepted articles, so please give us a few days to review your paper and let you know the next and final steps.

Lastly, if your institution or institutions have a press office, please let them know about your upcoming paper now to help maximize its impact. If they'll be preparing press materials, please inform our press team within the next 48 hours. Your manuscript will remain under strict press embargo until 2 pm Eastern Time on the date of publication. For more information, please contact onepress@plos.org.

You will receive an invoice from PLOS for your publication fee after your manuscript has reached the completed accept phase. If you receive an email requesting payment before acceptance or for any other service, this may be a phishing scheme. Learn how to identify phishing emails and protect your accounts at https://explore.plos.org/phishing.

If we can help with anything else, please email us at customercare@plos.org.

Thank you for submitting your work to PLOS One and supporting open access.

Kind regards,

PLOS One Editorial Office Staff

on behalf of

Dr. Giovanni Nassa

Academic Editor

PLOS One
